# A hybrid fungal growth and differential evolution algorithm for energy-efficient UAV trajectory planning in MEC

**DOI:** 10.1038/s41598-026-54288-4

**Published:** 2026-05-27

**Authors:** Alia A. Othman, Dina A. Elmanakhly

**Affiliations:** Department of Mathematics, Faculty of Science, Suez Canal University, Ismailia, 44745 Egypt

**Keywords:** Unmanned aerial vehicles, Mobile edge computing, Trajectory planning, Fungal growth optimizer, Optimized population size, Low complexity greedy, Engineering, Mathematics and computing

## Abstract

The deployment of Unmanned Aerial Vehicles (UAVs) in conjunction with Mobile Edge Computing (MEC) has come to be a viable approach to solve some challenges that face the internet of things systems, including energy consumption, latency, and data processing efficiency. However, trajectory planning optimization for UAVs in the MEC systems remains a challenging issue due to energy restrictions. This study introduces a trajectory planning algorithm, Fungal Growth–Differential Evolution (FGODE), seeking to minimize overall energy consumption without compromising task offloading efficiency and UAV mobility. The approach employs a hybrid optimization algorithm that combines the Fungal Growth Optimizer (FGO) and Differential Evolution (DE) algorithms to effectively maintain between searching new regions and refining promising solutions. The method also utilizes an optimized population size-based encoding mechanism to properly represent candidate solutions. Furthermore, a low-complexity greedy mechanism is employed to sequence the stop points along each UAV’s trajectory, while elite opposition-based learning and Gaussian mutation are utilized to accelerate convergence and mitigate premature stagnation. Several experiments have been conducted to compare with several algorithms. Experimental findings show that FGODE delivers more competitive results than state-of-the-art algorithms across several performance metrics, displaying higher optimization capability.

## Introduction

The Internet of Things (IoT) is a network of everyday physical items capable of communicating and transferring data, making a network without the participation of humans^1,2^. The aim of IoT is to improve connectivity and real-time applications and enhance delivery of new value across various industries^3^, such as manufacturing, transportation, energy, and agriculture. Examples include smart home devices^4^ that automate tasks like sending security alerts or adjusting cooler degrees, monitoring medical systems^5^, smart cities^6^, and smart agriculture^7^. As IoT evolves, its beneficial impacts are expected to expand, enabling a more interconnected, efficient, and responsive future for both enterprises and consumers. However, every advancement has inherent key limitations that must be mitigated to harness its complete value. These challenges include^8–10^ data management since these devices generate vast amounts of data daily, and the limitation is not only in storing this data but also in processing and analyzing it to extract insights^11^. Implementing advanced analytics, machine learning, and cloud solutions such as cloud computing^12,13^ and fog computing^14^ can help organizations manage IoT data more effectively^15,16^. Security risks and the interconnected nature of IoT systems indicate that a vulnerability in one device can compromise the entire network. This may lead to potential data breaches and unauthorized access. In addition, power consumption^17^ is one of the most important challenges in IoT, especially since these devices work on batteries with limited lifetimes. This limitation requires regular recharging or replacement of batteries, which raises maintenance costs and causes operational interruptions. So, studies tend to address these challenges by integrating Unmanned Aerial Vehicles (UAVs) with IoT technologies as a data-collecting tool^18^, enhancing the collection of energy-efficient data. UAVs serve as dynamic platforms for various applications^19^, including agricultural tasks^20^ such as crop spraying and irrigation, optimizing processes and resource use, and security domains like enabling effective airspace monitoring and exploration of hard-to-reach areas due to their portability and autonomy. Additionally, UAVs have gained popularity in disaster management and emergency response^21^ since they have emerged as vital tools in mitigating risks, aiding in rescues, and facilitating recovery efforts. UAVs play a key role in FANETs, where optimized routing is crucial. Bio-inspired algorithms have been widely applied for trajectory and routing optimization. A recent systematic review highlights these methods and their relevance for real-world UAV optimization problem as highlighted in^22^.

MEC technology offers enhanced computing and storage capabilities for devices, minimizing both energy use and latency^23,24^. However, MEC’s fixed infrastructure limits its adaptability to dynamic user demands^25^. Therefore, UAVs are employed to carry MEC servers, enabling mobility and improving service flexibility devices^26^. The integration of UAV-assisted MEC provides real-time processing, wide coverage, cost-effectiveness, and improved accuracy MEC^27–29^. Accordingly, efficient UAV trajectory planning becomes essential to minimize energy consumption during hovering, movement, and data transmission^30^. In this context, several studies have focused on UAV-assisted MEC optimization using deep learning techniques. For instance^31^, proposes an energy-efficient architecture based on Deep Deterministic Policy Gradient (DDPG) to jointly optimize UAV trajectory and bandwidth allocation. Similarly^32^, models the system as a partially observable Markov decision process and employs multi-agent deep reinforcement learning to optimize computation capacity and fairness. In addition^33^, introduces a decentralized multi-agent actor–critic approach for joint trajectory planning and task offloading. Furthermore^34^, proposes a Lagrange-based DDPG method for optimizing energy efficiency and delay. A dual-time-scale framework is introduced in^35^ to jointly handle resource allocation, offloading, and trajectory optimization, while^36^ addresses nonconvex resource allocation problems to improve system performance. Beyond trajectory optimization, recent works have explored advanced frameworks for resource allocation and offloading in edge environments. For example^37^, introduces a traffic-aware framework using prediction and deep reinforcement learning. Similarly, RoCoCache^38^ proposes a collaborative caching framework leveraging federated learning. In addition^39^, presents a federated learning approach for multi-edge load prediction. Furthermore, PFR-OA^40^ introduces a personalized federated reinforcement learning method for computation offloading, while^41^ proposes SR-CL combining deep reinforcement learning and convex optimization for service migration. Moreover, other studies focus on system-level frameworks and optimization strategies. For instance^42^, proposes a three-layer UAV-assisted MEC architecture. Recent works such as^43,44^, and^45^ explore graph-based learning and multi-agent reinforcement learning for adaptive resource management. Additionally^46^, employs Lyapunov-based optimization to enhance UAV trajectory and task coordination.

Among the most popular methods for resolving challenging optimization issues are metaheuristic algorithms, as they provide near-optimal solutions based on exploration and exploitation techniques without requiring precise mathematical derivation or prior knowledge of the system’s information^47^. These algorithms include techniques such as Particle Swarm Optimization (PSO), Genetic Algorithms (GA), Simulated Annealing (SA), Snow Ablation Optimizer (SAO)^48^, and others. The main categories of metaheuristic algorithms relevant to UAV and MEC optimization can be summarized as shown in Table 1. Recent metaheuristic algorithms include Snow Ablation Optimizer (SAO^48^, which mimics snow sublimation to balance exploration and exploitation; hybrid grasshopper optimization algorithm (HGOA)^49^, a hybrid grasshopper-butterfly algorithm with local search; Multi-strategy improved Slime Mould Algorithm (MSMA)^50^ and an enhanced slime mould algorithm called AGSMA^51^, improved slime mould algorithms with adaptive search and mutation; QLJAYA^52^, which integrates Q-learning and gradient search for better convergence; and ESLPSO^53^, an enhanced social learning particle swarm optimizer for robust PV parameter estimation. In addition, DEF-SAC^54^ employs a multi-mutation strategy with self-adaptive configuration and diversity-aware initialization to effectively handle high-dimensional optimization problems.

**Table 1:** Representative metaheuristic algorithms for optimization.

Category	Algorithm
Swarm-based	Artificial Protozoa Optimizer^55^
	Sperm Swarm Optimization^56^
Physical-based	Chernobyl Disaster Optimizer^57^
	Bermuda Triangle Optimizer (BTO)^58^
Manmade-based	Five Phases Algorithm (FFA)^55^

Several advanced variants of Differential Evolution (DE) have been proposed to enhance optimization performance and address limitations such as premature convergence and parameter sensitivity^59^. For instance, LSHADE-Code^60^ introduces adaptive parameter control and novel mutation strategies to improve exploration and convergence stability. Similarly, AHDE-LS^61^ integrates DE with adaptive local search mechanisms, combining global exploration and local exploitation while dynamically adjusting control parameters to enhance efficiency and robustness. However, several recent studies have raised concerns regarding the lack of scientific rigor and true novelty in many of these approaches^62^. Component-based analyses have shown that many such algorithms, including WOA, AOA, and ChOA, often reuse existing mechanisms under different terminologies without introducing fundamentally new concepts^63^. Moreover, these methods may suffer from structural biases and design flaws, limiting their performance, particularly on shifted or complex benchmark problems^64,65^. These observations highlight the need for more carefully designed and well-justified optimization frameworks. To overcome these limitations, several works have applied metaheuristics to UAV-MEC optimization problems. In^66^ a SA algorithm is employed to reduce energy consumption of UAVs during transmission tower inspections. Along with^67^, this study investigates joint task scheduling and edge caching in MEC to address challenges caused by high user mobility. A hybrid algorithm combining PSO and GA is proposed, significantly improving task completion rates, cache hit ratios, and load balancing compared to classical and recent hybrid intelligent algorithms. Similarly^68^, adapts number of the recently published metaheuristic algorithms, such as Spider Wasp Optimizer (SWO), Generalized Normal Distribution Optimization (GNDO), and Gradient-Based Optimizer (GBO) designed to optimize UAV deployments and achieve minimal overall energy consumption. In^69^ addresses the energy limitation problem in wireless sensor networks. To enhance performance, the authors optimized routing, clustering, node placement, coverage, and data aggregation using GA. Simulation results demonstrated that the proposed approach could reduce energy consumption by up to 50%.

In UAV trajectory optimization, various approaches have been proposed. For example^70^, some methods focus on decomposing the population into poor and elite solutions to reduce energy consumption and extend network lifetime. Others adopt hybrid metaheuristic strategies that jointly optimize UAV flight paths, task assignments, and resource distribution, as in the SHADE-NE approach^71^, Which leverages adaptive parameter control to balance exploration and exploitation. EPSCA^72^ is an enhanced Sine Cosine Algorithm that balances exploration and exploitation using elite pool strategy, Brownian motion, local search, and mutation, showing strong performance on benchmark problems and PV system optimization. Bi-criterion Ant Colony Optimization frameworks^73^ aim to simultaneously minimize execution time and operational costs by employing multiple heterogeneous colonies, each dedicated to a specific optimization objective and using structured pheromone matrices to construct feasible solutions. Additionally, multi-UAV edge computing scheduling algorithms such as MUECRS^74^ address mixed discrete-continuous decision spaces by optimizing trajectories, execution strategies, and resource allocation simultaneously. Along with this^75^, proposes a Lyapunov optimization method to reduce combined energy cost in user terminals governed by adjustable backlog queues. Alternating metaheuristic optimization algorithms are applied to optimize transmit power, bandwidth allocation, and UAV trajectories, complemented by a two-stage UAV operating scheme. Moreover^76^, reviews metaheuristic algorithms for node placement in UAV Communication Networks (UAVCN), focusing on maximizing coverage and connectivity while minimizing latency and energy consumption. Selected hybrid algorithms are evaluated using computational time and coverage metrics, demonstrating their effectiveness in multi-objective optimization and providing guidance for future UAVCN deployments. This paper^77^ introduces various multiobjective trajectory planning algorithms (MTPA) and is grounded in multiple metaheuristic approaches that utilize adaptive population size and the principles of optimal compromise solutions. Furthermore, the paper proposes a new mechanism known as the cyclic selection mechanism (CSM) to effectively regulate the population size, simultaneously optimizing both the number of stopping points and the maximum allowable function evaluations. In^78^ a new algorithm called ITPA-GBOKM is proposed. It integrates a transfer-based encoding method, a GBO, and the K-Medoids clustering algorithm to improve solution accuracy, energy efficiency, and UAV- stop point associations. K-Medoids is chosen for its robustness against outliers, while the GBO efficiently searches for energy-saving stop points configurations. In the context of the limited theoretical support for metaheuristic algorithms (MAs), recent studies have explored advanced frameworks to better understand their behavior. In particular, complex network theory has been used to model population interactions and reveal key properties such as small-world structures and interaction patterns^79,80^. This perspective provides valuable insights into the dynamics of evolutionary algorithms and supports the development of more effective optimization methods. Despite the extensive research on UAV-assisted MEC systems, several limitations still exist. First, many existing approaches rely on deep reinforcement learning or optimization frameworks that focus mainly on resource allocation and task offloading, while giving limited attention to efficient UAV trajectory planning under dynamic and energy-constrained environments. Second, although metaheuristic algorithms have been widely applied for trajectory optimization, many recent methods suffer from issues such as premature convergence, lack of balance between exploration and exploitation, and limited generalization due to their dependence on specific problem settings. In addition, several metaphor-based algorithms introduce new terminologies without significant methodological improvements, which may affect their robustness and practical applicability. Furthermore, existing hybrid optimization approaches often combine multiple techniques without clearly addressing the interaction between trajectory design, stop point selection, and resource utilization in UAV-assisted MEC systems. Therefore, there is a need for a more effective and well-structured optimization framework that can jointly handle trajectory planning while maintaining computational efficiency and robust search performance.

To conclude, this research provides several noteworthy improvements to the field of UAV-assisted MEC systems. These contributions are outlined as follows:

- We propose a novel trajectory planning algorithm, termed Fungal Growth–Differential Evolution (FGODE), to address the Trajectory Planning Optimization (TPO) problem in UAV-assisted MEC systems. The proposed method integrates several key components:
- We develop a hybrid optimization framework that integrates the exploration strength of the Fungal Growth Optimizer (FGO) with the exploitation capability of Differential Evolution (DE) to achieve a balanced and efficient search mechanism.
- We utilize an optimized population size-based encoding strategy (oPS) for efficient regulation of UAV stop points.
- We apply a Low-Complexity Greedy (LCG) approach for generating the most efficient trajectory for each UAV.
- Leveraging Gaussian Mutation (GM) to improve convergence speed and escape local optima.
- Evaluating the performance of FGODE across eight instances with IoT devices ranging from 60 to 200, demonstrating its adaptability to small and medium-scale deployment scenarios.
- Comparing the proposed algorithm with several Advanced methods verified using various performance indicators to ensure effectiveness and robustness.
- The results of the experiments reveal that FGODE consistently delivers superior and statistically significant performance compared to competing approaches.

The paper is divided into the following parts: Sect. 2 outlines the problem formulation; Sect. 3 presents the background algorithms; Sect. 4 describes the proposed algorithms; Sect. 5 presents the experimental setup; Sect. 6 examines the findings; and Sect. 7 summarizes with insights and opportunities for further investigation.

## Problem formulation

**Fig. 1:**
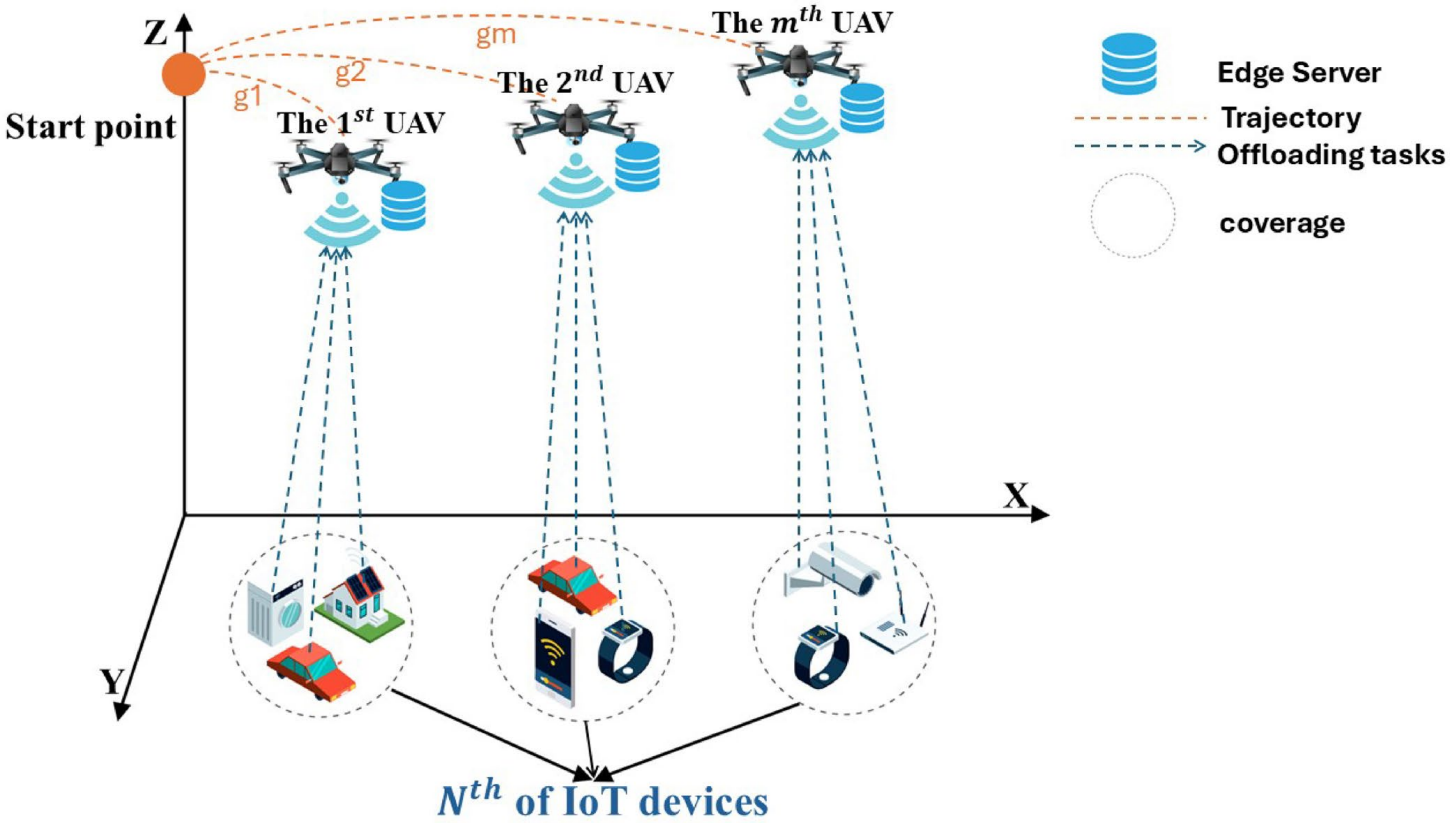
Multi-UAV-assisted MEC system. The figure was drawn using Adobe Illustrator 2020 (www.adobe.com/products/illustrator).

In Fig. 1, multi-UAV-supported MEC systems with n IoT devices and m UAVs is shown. This system’s collection of IoT devices is symbolized as N = {1, 2,., n}, and UAVs with MEC servers are symbolized as M = {1, 2,., m}. Each task needs to be processed, transmitted to the MEC server and subsequently received back by the IoT devices. Each UAV follows a trajectory defined by a set of stop points $\:{\mathrm{K}}_{\mathrm{i}}=\left\{\mathrm{1,2},\:\dots\:,{\mathrm{k}}_{\mathrm{i}}\right\}$, which it traverses to gather and handle information from IoT devices. The trajectory for the ith UAV is represented as $\:{\mathrm{g}}_{\mathrm{i}}=\left\{\left({\mathrm{X}}_{\mathrm{i}}^{\mathrm{l}},{\mathrm{Y}}_{\mathrm{i}}^{\mathrm{l}},{\mathrm{H}}_{\mathrm{i}}^{\mathrm{l}}\right)\right\}$, where l ∈ $\:{\mathrm{k}}_{\mathrm{i}}$ and $\:{\mathrm{H}}_{\mathrm{i}}^{\mathrm{l}}$ is the height of the ith UAV for each stop point, with a fixed value assigned as represents in^81^.

The following formula yields the distance between the jth device, which is situated at $\:\left({\mathrm{x}}_{\mathrm{j}},{\mathrm{y}}_{\mathrm{j}},0\right)$, and the ith stop point:

1 $$\:{d}_{jil}=\sqrt{{\left({X}_{i}^{l}-{x}_{j}\right)}^{2}+{\left({Y}_{i}^{l}-{y}_{j}\right)}^{2}+{H}_{i}^{2}}$$

To facilitate the assignment of tasks to UAVs, we introduce a binary variable $\:{a}_{jil}$. This variable indicates whether the jth IoT device transmits its task to the ith UAV at the lth stop point. The following formula is used to mathematically assign this variable a value of 1 or 0:

2 $$\:{C}_{1\:}:\:\:\:{a}_{jil}=\left\{\begin{array}{c}1,if\left(i,\:l\right)=\mathrm{arg}\underset{i\in\:M,\text{}l\in\:{K}_{j}}{\mathrm{min}}{d}_{jil,}\:\:\:\\\:0,\:\:\:\:\:\:\:\:\:\:\:\:otherwise\end{array}\right.$$

Each jth task is exclusively assigned to a single UAV at a single stop point thanks to the following constraint:

3 $$\:{C}_{2\:}:\sum\:_{i=1}^{m}\sum\:_{l=1}^{{k}_{i}}{a}_{jil}=1,\:\:\forall\:j\in\:N\:$$

Because of bandwidth-limited capacity, the ith UAV at the lth stop point cannot serve more than M IoT devices. As an outcome of this limitation, the jth UAV at the lth stop point is only capable of concurrently processing the tasks of δ IoT devices. It is necessary to add the following formula to meet the restrictions in order to guarantee that this limitation is considered:

4 $$\:{C}_{3\:}:\:\:\:\:\sum\:_{j=1}^{n}{a}_{jil}\le\:\delta\:,\:\forall\:i\in\:M,\:\forall\:l\in\:{K}_{i}$$

Additionally, at least one IoT device is served by each UAV positioned at each stop point. Consequently, the total count of stop points for all UAVs, represented by *k*, must comply with the following restriction:

5 $$\:{C}_{4}\::\:\:\:{k}_{min}\le\:k\le\:{k}_{max}\left|{k}_{min}=\lfloor\frac{n}{M}\rfloor\&{\:k}_{max}=n\:\right.$$

The power gain of the communication channel linking the jth IoT device with the ith UAV at the lth stop point when data is being transmitted can be modeled using the listed below equation:

6 $$\:{G}_{ijl}={G}_{0}{d}_{jil}^{-2}=\frac{{G}_{0}}{{\left({X}_{i}^{l}-{x}_{j}\right)}^{2}+{\left({Y}_{i}^{l}-{y}_{j}\right)}^{2}+{H}_{i}^{2}}$$

Where $\:{G}_{0}$ is the Reference channel gain and to determine the rate of transfer of data from the jth IoT device to the ith UAV at the lth stop point, the subsequent mathematical formula may be employed:

7 $$\:{R}_{jil}={Blog}_{2}\left(1+\frac{{P}_{j}{G}_{jil}}{{\beta\:}^{2}}\right),\:\forall\:j\in\:N,\:i\in\:M,\:l\in\:{K}_{i}$$

Here, B represents the bandwidth, $\:{P}_{j}$ denotes the power of transmission of the jth IoT device, and $\:{\beta\:}^{2}$corresponds to the power of the additive white Gaussian noise. The amount of energy and time used by the jth IoT device for data transmission to the ith UAV at the lth stop point are derived as follows:

8 $$\:{T}_{jil}^{iot}=\frac{{D}_{j}}{{R}_{jil}},\:\forall\:j\in\:N,\:i\in\:M,\:l\in\:{K}_{i}$$

9 $$\:{E}_{jil}={P}_{i}{T}_{ijl}^{iot}=\frac{{{P}_{j}D}_{j}}{{R}_{jil}},\:\forall\:j\in\:N,\:i\in\:M,\:l\in\:{K}_{i}$$

The overall energy consumption of all IoT devices is represented by^81^:

10 $$\:F1={E}_{iot}=\sum\:_{j=1}^{n}\sum\:_{i=1}^{m}\sum\:_{l=1}^{{k}_{j}}{b}_{jil}{E}_{jil}$$

Once UAVs receive the necessary input data, they begin processing their assigned tasks. The time required for the ith UAV at the lth stop point to complete the jth task, given the computing resource $\:{\mathrm{c}}_{\mathrm{j}\mathrm{i}\mathrm{l}}$, can be determined using the formula that follows:

11 $$\:{T}_{jil}^{u}=\frac{{S}_{j}{D}_{j}}{{c}_{jil}},\:\forall\:j\in\:N,\:i\in\:M,\:l\in\:{K}_{j}$$

The ith UAV stays at the lth stop point until it completes all its assigned tasks, after which it proceeds to the next stop point. As a result, the total hovering time of the ith UAV at the lth stop points corresponds to the cumulative time needed to complete all allocated tasks. The duration of the ith UAV’s hover at the lth stop point can be approximated as follows:

12 $$\:{T}_{jl}^{u}=\underset{j\in\:N}{\mathrm{max}}\left\{{a}_{jil}\left({T}_{jil}^{u}+{T}_{jil}^{iot}\right)\right\}$$

Following this, The hovering energy of the ith UAV at the lth stop point is given by:

13 $$\:{E}_{i}^{u}={\sum\:}_{l=1}^{{k}_{i}}{p}^{u}{T}_{il}^{u},\:\forall\:i\in\:M$$

where $\:{p}^{u}$ represents the power consumed by the UAV while hovering. The ith UAV’s time and energy required to move through all stop points along its generated trajectory $\:{g}_{i\:}$can be calculated as follows:

14 $$\:{T}_{i}^{F}=\frac{1}{v}\sum\:_{l=2}^{{k}_{i}}\sqrt{{\left({X}_{i}^{l}-{X}_{i}^{l-1}\right)}^{2}+{\left({Y}_{i}^{l}-{Y}_{i}^{l-1}\right)}^{2}+{H}_{i}^{2}}\:,\:\forall\:i\in\:M\:$$

15 $$\:{E}_{i}^{F}={p}^{F}{T}_{i}^{F},\:\forall\:i\in\:M$$

While $\:{p}^{F}$represents the flight power, and $\:v$ denotes the speed of the UAV’s flight. Overall energy usage is influenced by both the energy used during hovering and flying, and it can be calculated as detailed below:

16 $$\:F2={E}_{uav}=\sum\:_{i=1}^{m}\left({E}_{i}^{F}{+E}_{i}^{u}\right)$$

This study introduces a TPO algorithm designed to optimize the flight paths of UAVs within a multi-UAV-assisted MEC system. The primary goal is to reduce the overall energy usage of IoT devices and UAVs. In general, presented below is a mathematical formulation of the optimization problem:

$$\:\underset{{\mathcal{g}}_{1},{\mathcal{g}}_{2},\:\dots\:,\:{\mathcal{g}}_{m}}{\mathrm{min}}\:\:\:\:F=F1+\sigma\:F2$$

$$\:S.t.\:\:\:{C}_{1\:}:\:\:\:{a}_{jil}=\left\{\begin{array}{c}1,if\left(i,\:l\right)=\mathrm{arg}\underset{i\in\:M,{\:\:l}\in\:{K}_{i}}{\mathrm{min}}{d}_{jil,}\\\:0,\:otherwise\:\:\:\:\:\:\:\:\:\:\:\:\:\:\:\:\:\:\:\:\:\:\:\:\:\:\end{array}\right.$$

$$\:{C}_{2\:}:\sum\:_{i=1}^{m}\sum\:_{l=1}^{{k}_{i}}{a}_{jil}=1,\:\:\forall\:j\in\:N$$

$$\:{C}_{3\:}:\:\:\:\:\sum\:_{j=1}^{n}{a}_{jil}\le\:\delta\:,\:\forall\:i\in\:M,\:\forall\:l\in\:{K}_{i}\:$$

$$\:{C}_{4}\::\:\:\:{k}_{min}\le\:k\le\:{k}_{max}$$

$$\:{C}_{5}\::\:\:\:{X}_{min}\le\:{X}_{i}^{l}\le\:{X}_{max}\:,\forall\:i\in\:M,\:\forall\:l\in\:{K}_{i}$$

$$\:{C}_{6}\::\:\:\:{Y}_{min}\le\:{Y}_{i}^{l}\le\:{Y}_{max\:\:},\forall\:i\in\:M,\:\forall\:l\in\:{K}_{i}$$

where $\:\sigma\:$ is a weighting factor positively valued, ensuring that the effect of F2 on the objective function is balanced against F1. Here, $\:{X}_{max\:\:}$and $\:{X}_{min}$ represent the maximum and minimum bounds of $\:{X}_{i}^{l}$, respectively, while $\:{Y}_{max\:}$ and $\:{Y}_{min}$ denote the maximum and minimum bounds of $\:{Y}_{i}^{l}$.

## Background algorithms

### Fungal growth behavior

FGO is a new metaheuristic algorithm inspired by nature^82^ based on the growth behaviors of fungi. Fungi play a critical role in nutrient cycling and exhibit unique growth behaviors, such as hyphal extension, branching, and spore germination. These behaviors are harnessed in FGO to enhance optimization processes. Specifically, each biological behavior is mapped to a corresponding optimization operator that controls either exploration or exploitation within the search space.

### Hyphal tip growth

The algorithm mimics hyphal growth by utilizing chemotropism to navigate and explore the search space effectively, allowing it to determine and take advantage of nutrient-rich areas. In optimization terms, this mechanism corresponds to adaptive solution updating, where candidate solutions are guided either toward promising regions (exploitation) or diversified to explore new areas (exploration). This dynamic approach helps prevent stagnation in local optima and accelerates the rate of convergence. The mathematical model is separated into two phases. The first is the exploration phase. If the normalized probability value)pi), which is computed using the relative fitness of the $\:i$th solution, is smaller than the exploration rate (Er), then the solution updates by (18) as follows:

18 $$\:{\overrightarrow{S}}_{i}^{t+1}={\overrightarrow{S}}_{i}^{t}+E\cdot\:\overrightarrow{D}$$

Where $\:{\overrightarrow{S}}_{i}^{t}$ is the current position (solution) of the ith hypha at iteration t, E is the growth rate dependent on fitness value and exploration factor, $\:\overrightarrow{D}$ direction vector formed according to the difference between two random solutions from the population and updated the position of the hypha. Otherwise, the second phase takes place, the exploitation phase represents intensification around high-quality solutions, where the search is directed using the best-so-far solution or guided directions to refine candidate solutions. the solution is update using (21) as described:

19 $$\:{\overrightarrow{S}}_{i}^{t+1}={\overrightarrow{S}}_{i}^{t}+{\eta\:}_{i}\cdot\:{\overrightarrow{D}}_{i}^{e2}+\overrightarrow{{E}_{c}}\cdot\:\left[{r}_{1}>{r}_{2}\right],$$

20 $$\:{\overrightarrow{S}}_{i}^{t+1}={\overrightarrow{S}}_{i}^{t}+{\eta\:}_{i}\cdot\:{\overrightarrow{D}}_{i}^{e}+\overrightarrow{{E}_{c}}\cdot\:\left[{r}_{3}>{E}_{P}\right],$$

21 $$\:{\overrightarrow{S}}_{i}^{t+1}=\left\{\begin{array}{c}Second\:state\left(19\right),{r}_{4}>{r}_{5}\:\\\:First\:state\:\left(20\right),\:else\end{array}\:,i=1,\:2,\dots\:,N,\right.$$

Where $\:{r}_{1},\dots\:,{r}_{5}$ are random numbers in [0, 1], $\:{\eta\:}_{i}$ is the normalized growth rate, $\:{\overrightarrow{D}}_{i}^{e2}$ is the growth direction away from the current best solution (opposite chemotropism, simulates avoiding harmful areas) and $\:\overrightarrow{{E}_{c}}$ is an extra exploratory component (randomized vector based on difference of two solutions) and $\:{\overrightarrow{D}}_{i}^{e}$ is the growth direction vector towards either for random solution or the best so far solution (nutrient-rich) and $\:{E}_{P}$ is the probability controlling randomness level of environmental reaction.

### Hyphal branching

The branching behavior of hyphae is also replicated. From an optimization perspective, this operator introduces controlled perturbations across solution dimensions, allowing partial updates while preserving some original values, thereby enhancing diversity without disrupting solution stability, facilitating exploration of surrounding areas for better nutrient acquisition, thereby enriching the optimization process. It is mathematically described as follows:

22 $$\:{S}_{ij}^{t+1}=\left[{R}_{j}>{r}_{6}\right]\cdot\:{S}_{ij}^{t}+\left(1-\left[{R}_{j}>{r}_{6}\right]\right)\cdot\:\left({S}_{ij}^{t}+{r}_{7}\cdot\:{E}^{L}\cdot\:{S}_{ij}^{ep1}+\left(1-{r}_{7}\right)\cdot\:{E}^{L}\cdot\:{D}_{ij}^{ep2}\right),\:j=1,\:2,\dots\:,d,$$

Where $\:\left[{R}_{j}>{r}_{6}\right]$ is used to retain original dimension value and $\:{E}^{L}$ growth rate for lateral branching.

### Spore germination

Spore germination is incorporated to enhance the exploitation capability of the algorithm by guiding the search toward promising regions. As the optimization process progresses, spores are increasingly generated around the best-so-far solution, allowing the algorithm to intensify the search in high-quality areas. This mechanism helps refine candidate solutions and accelerates convergence toward near-optimal solutions while maintaining controlled diversity.

23 $$\begin{aligned}\:{\mathbf{S}}_{\mathbf{i},\mathbf{j}}^{\mathbf{t}+1}=\left[{\mathbf{R}}_{\mathbf{j}}<{\mathbf{r}}_{6}\right]\cdot\:{\mathbf{S}}_{\mathbf{i}\mathbf{j}}^{\mathbf{t}}+\left(1-\left[{\mathbf{R}}_{\mathbf{j}}<{\mathbf{r}}_{6}\right]\right)\\\cdot\:\left(\frac{\left(\frac{\left(\left(\frac{\mathbf{t}}{{\mathbf{t}}_{\mathbf{m}\mathbf{a}\mathbf{x}}}\right)\cdot\:{\mathbf{S}}_{\mathbf{j}}^{\mathbf{*}}+\left(1-\frac{\mathbf{t}}{{\mathbf{t}}_{\mathbf{m}\mathbf{a}\mathbf{x}}}\right)\cdot\:{\mathbf{S}}_{\mathbf{a},\mathbf{j}}^{\mathbf{t}}\right)}{2}+{\overrightarrow{\mathbf{S}}}_{\mathbf{b}}^{\mathbf{t}}\right)}{2}+{\mathbf{S}}_{\boldsymbol{g}}\cdot\:{\mathbf{r}}_{7}\cdot\:\mathbf{E}\cdot\:\left|\frac{\left({\mathbf{S}}_{\mathbf{c},\mathbf{j}}^{\mathbf{t}}+{\mathbf{S}}_{\mathbf{a},\mathbf{j}}^{\mathbf{t}}+{\mathbf{S}}_{\mathbf{b},\mathbf{j}}^{\mathbf{t}}\right)}{3}-{\mathbf{S}}_{\mathbf{i},\mathbf{j}}^{\mathbf{t}}\right|\right),\:\mathbf{j}=1,\:\dots\:,\mathbf{d},\:\end{aligned}$$

Where $\:{\mathrm{S}}_{\mathrm{g}}$ is a random selection of either 1 or −1 and $\:{\mathrm{S}}_{\mathrm{c}}^{\mathrm{t}},\:\:{\mathrm{S}}_{\mathrm{a}}^{\mathrm{t}},\:{\:\mathrm{S}}_{\mathrm{b}}^{\mathrm{t}}$ are three randomly selected solutions. The trade-off between spore germination and lateral branching in the suggested FGO is realized with a probability of 0.5, as described in (24) and The FGO algorithm’s pseudocode is outlined in **Algorithm 1.**

24 $$\:{\overrightarrow{S}}_{i}^{t+1}=\left\{\begin{array}{c}Apply\left(22\right),{r}_{8}<0.5\:\\\:Apply\:\left(23\right),\:else\end{array}\:,i=1,\:2,\dots\:,N,\:\:\:\right.$$

**Algorithm 1:**
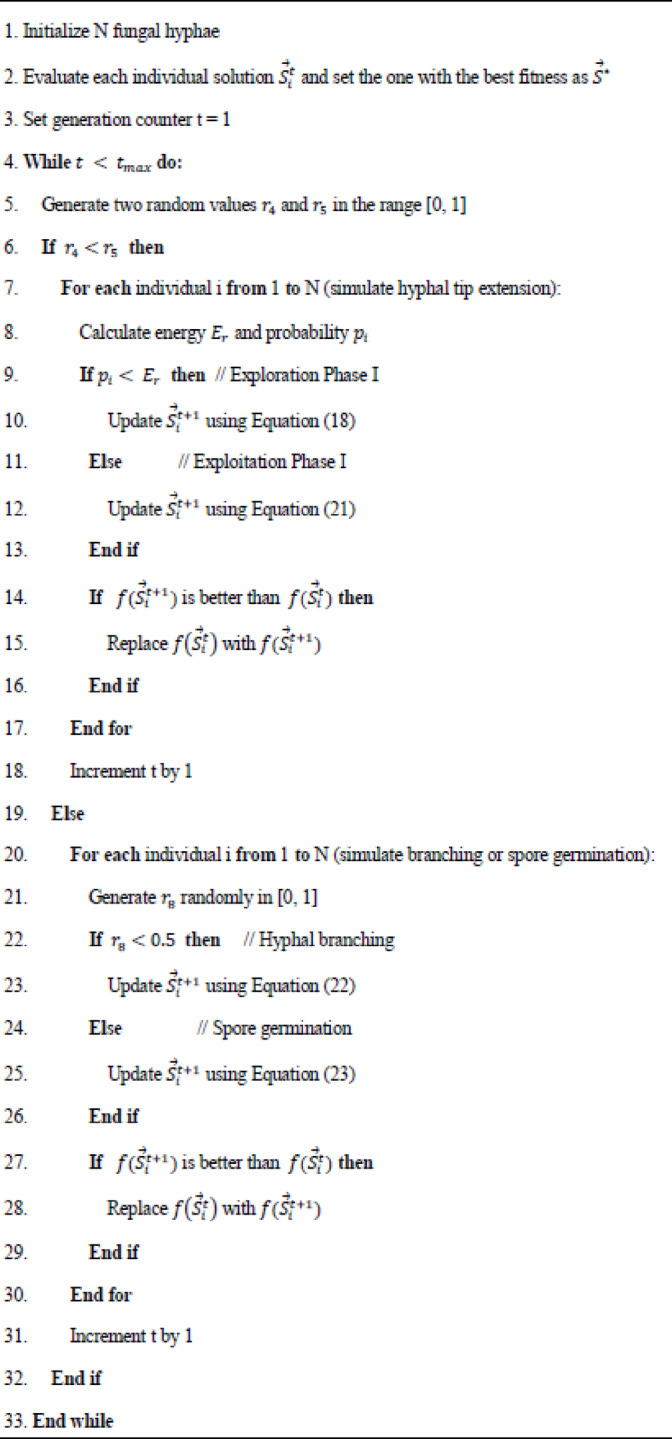
Pseudocode for FGO.

### Differential evolution

DE^83^ is a population-based optimization algorithm inspired by the mechanisms of natural evolution. It is particularly effective to handle nonlinear and continuous optimization scenarios. Like Genetic Algorithms, DE operates through three fundamental processes: **mutation**, **crossover**, and **selection**. The algorithm’s pseudocode is outlined in **Algorithm 2**.

### Mutation operator

In this step, a new candidate solution called the mutant vector $\:{\vec{v}}_{i}^{t}$is generated for each target vector $\:{\vec{x}}_{i}^{t}$. The goal of this operator is to increase population diversity to facilitate more effective exploration of the search space. Among the different mutation strategies, such as “DE/best/1” and “DE/rand/1”, the present study employs the “DE/rand/1” scheme due to its strong exploitation ability and effective refinement of promising regions within the search space. The formulation of the process is given by:

25 $$\:{\vec{v}}_{i}^{t}={\vec{x}}_{a}^{t}+F\times\:({\vec{x}}_{k}^{t}-{\vec{x}}_{j}^{t})$$

where $\:a,k,j\:$representing three unique indices that are chosen at random within the population, and $\:F\:$is a scaling factor typically ranging within the range [0, 1].

### Crossover operator

The crossover phase generates a *trial vector* $\:{\vec{u}}_{i}^{t}$ by combining the elements of $\:{\vec{x}}_{i}^{t}$and $\:{\vec{v}}_{i}^{t}$according to a crossover probability $\:Cr$. This operation ensures that each new individual inherits information from both parents. It is defined as:

26 $$\:{u}_{i,j}^{t}=\left\{\begin{array}{ll}{v}_{i,j}^{t},&\:\mathrm{if\:}\left(rand\le\:Cr\right)\mathrm{\:or\:}\left(j={j}_{r}\right)\\ \\\:{x}_{i,j}^{t},&\:\mathrm{otherwise}\end{array}\right.\:$$

where $\:{j}_{r\:}$ is an index selected at random to guarantee one or more parameters are taken from the mutant vector.

### Selection operator

Lastly, the selection process determines whether the trial vector $\:{\vec{u}}_{i}^{t}$ replaces the current solution $\:{\vec{x}}_{i}^{t}$. If the new candidate provides a better fitness value, it survives for the next generation; alternatively, the original solution is retained. The process is modeled as:

27 $$\vec{x}_{i}^{t} = \begin{cases}\vec{u}_{i}^{t}, & \text{if } f\left(\vec{u}_{i}^{t}\right) < f\left(\vec{x}_{i}^{t}\right) \\\vec{x}_{i}^{t}, & \mathrm{otherwise}\end{cases}$$

**Algorithm 2:**
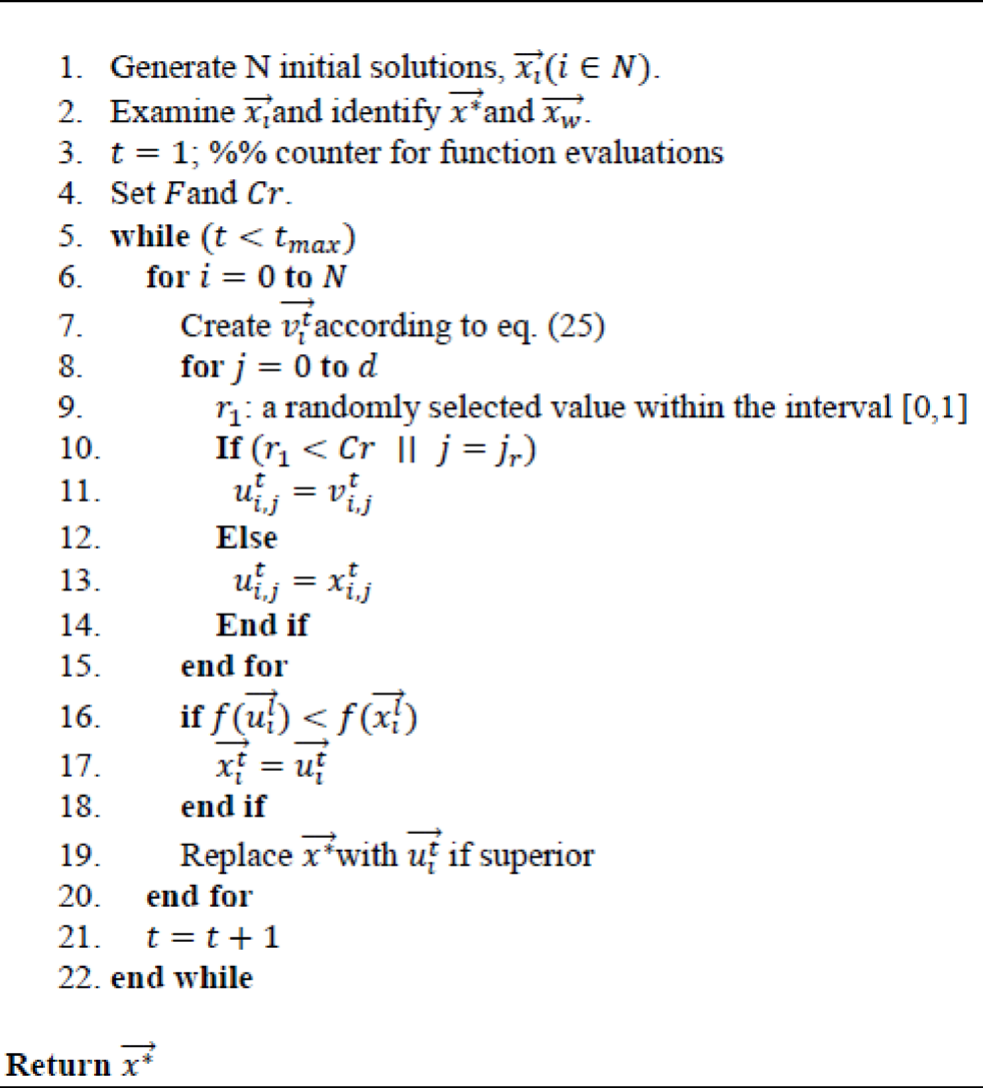
Standard DE.

### Proposed hybrid FGODE algorithm

Within this section the procedures employed to develop a hybrid optimization algorithm based on the FGO, which is further enhanced with DE operators to efficiently perform the optimization challenges in multi-UAV-supported MEC systems. The process contains the following steps:

1. During the initialization step, a varied population of potential solutions is produced to establish a strong foundation for the algorithm.
2. The oPS mechanism is applied to effectively represent solutions within the optimization problem space.
3. Exploration mechanisms, where Gaussian Mutation (GM) is incorporated to enhance population diversity and introduce controlled stochastic variations that improve global search capability.
4. The Exploitation mechanisms, where DE is employed for local refinement, and the LCG mechanism serve to prioritize the UAV-assigned stop point.
5. The pseudocode detailing the proposed FGODE algorithm.

The motivation behind adopting the proposed FGODE algorithm stems from the need to effectively balance exploration and exploitation in complex optimization problems such as UAV trajectory planning in MEC systems. FGO is selected due to its biologically inspired mechanisms, including hyphal extension, branching, and spore germination, which provide strong exploration capabilities and help maintain population diversity. However, FGO may exhibit relatively slow convergence in later stages due to its stochastic nature. On the other hand, DE is well-known for its efficient exploitation ability, fast convergence speed, and strong local search performance, although it may suffer from premature convergence in complex search spaces. Therefore, integrating FGO with DE enables the proposed FGODE algorithm to leverage the complementary strengths of both approaches, achieving a more balanced and efficient search process. This hybridization enhances solution quality, accelerates convergence, and improves robustness in solving UAV trajectory optimization problems under dynamic MEC environments.

### Initialization step

The optimization process starts by initializing the population so a three-dimensional matrix $\:\overrightarrow{{X}_{t}}\in\:{\mathbb{R}}^{{N}_{SP}\times\:3}$ is generated using a hybrid initialization strategy to ensure population diversity and spatial coverage. Here, $\:{N}_{SP}$ denotes the total number of stop points assigned to UAVs, and the three dimensions represent the UAV coordinates $\:[X,Y,H]$. The proposed hybrid method integrates three complementary mechanisms: Random Initialization, Elite Opposition-Based Learning (EOBL), and Low-Density Region Exploration (LDRE). The random part of the population is generated according to the equation below:

28 $$\:{\overrightarrow{X}}_{rand,i}=\overrightarrow{L}+\overrightarrow{r}\circ\:(\overrightarrow{U}-\overrightarrow{L}),i=\mathrm{1,2},...,\frac{{N}_{SP}}{2}$$

Where $\:\overrightarrow{L}=\left[{X}_{min},{Y}_{min},{H}_{min}\right]\:$and $\:\overrightarrow{U}=\left[{X}_{max},{Y}_{max},{H}_{max}\right]\:$represent the minimum and maximum constraints for each dimension, and $\:\overrightarrow{r\:}$is a random uniformly distributed vector in [0,1].

EOBL^84^ is employed. Within this mechanism, elite individuals selected using randomly generated population are used as guided references to generate opposition solutions. These elite-guided opposite candidates are constructed to symmetrically explore unexplored regions of the search space within the predefined boundaries, thereby improving population diversity and providing a high-quality starting distribution. The opposition population is generated as:

29 $$\:{\overrightarrow{X}}_{EOBL,i}=\overrightarrow{U}+\overrightarrow{L}-{\overrightarrow{X}}_{rand,i}$$

After combining both $\:{\overrightarrow{X}}_{rand}$and $\:{\overrightarrow{X}}_{OBL}$, the LDRE process identifies individuals in low-density regions based on Euclidean distances and slightly perturbs them using Gaussian noise to enhance exploration:

30 $$\:{\overrightarrow{X}}_{LDRE,i}={\overrightarrow{X}}_{low,i}+\sigma\:\cdot\:\mathcal{N}\left(\mathrm{0,1}\right)$$

Finally, the complete hybrid-initialized population is formed as:

31 $$\:\overrightarrow{{X}_{t}}=[{\overrightarrow{X}}_{rand};{\overrightarrow{X}}_{EOBL};{\overrightarrow{X}}_{LDRE}]$$

and truncated to size $\:{N}_{SP}$if necessary.

### Encoding mechanisms in planning trajectory optimization problems

**Fig. 2:**
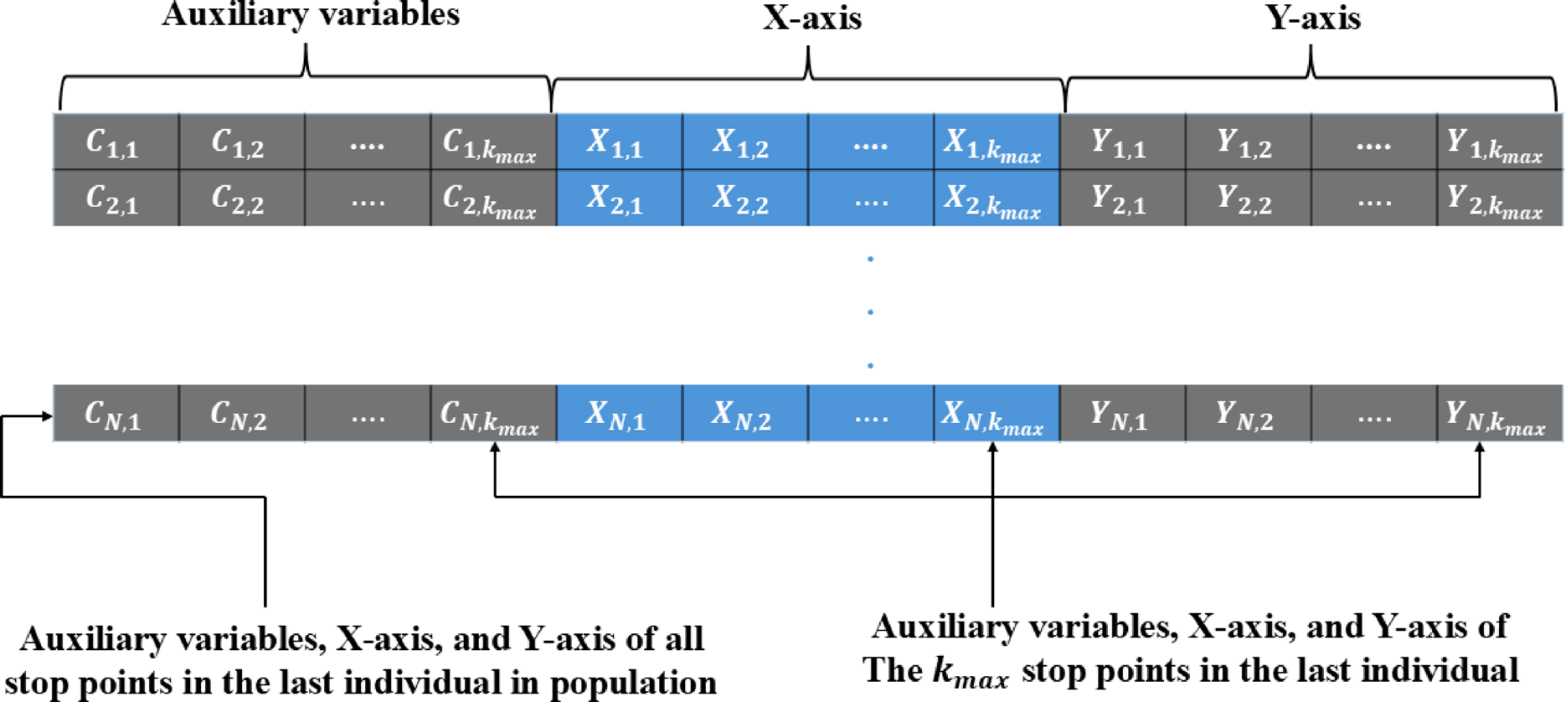
TPO solution representation under auxiliary variable-based encoding mechanism.

Two key encoding schemes for optimizing UAV TPO problem. The first method, called the Auxiliary Variable-Based Encoding Mechanism (see Fig. 2), assigns each individual in the population the responsibility of managing multiple stop points, where each stop point includes three parts: the first and the second represent x and y coordinates, and the third part holds a binary variable indicating the determination of whether a candidate stop point is incorporated into the deployment plan and assigned for operational use or not. This results in a solution length of 3k, where k is the UAV stop point count. As the dimensions of each solution increase, the efficiency of the metaheuristic algorithms tends to degrade. This encoding strategy results in inefficient use of memory since it presents difficulties since it requires every individual to account for all stop points, even those that were not chosen for deployment.

**Fig. 3:**
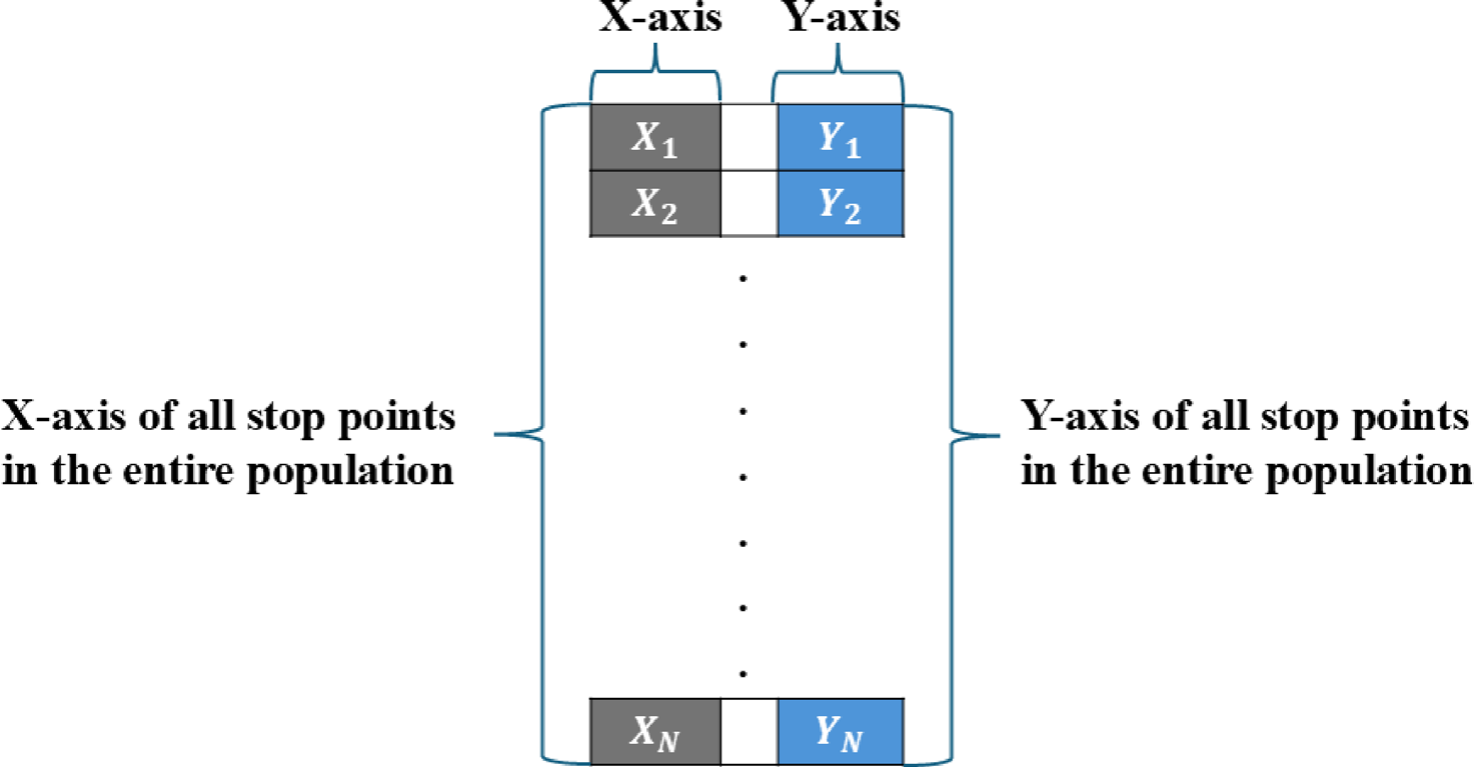
TPO solution representation under a VIPS.

In response to these limitations^81^, introduced a more effective approach known as The Variable Population Size-Based Encoding (VIPS) Mechanism (see Fig. 3), which is designed by eliminating auxiliary variables and maintaining a constant dimensionality across solutions. It focuses on determining the optimal count and locations of stop points aimed at energy minimization for each UAV. The mechanism employs three operations: insertion, removal, and replacement, creating three distinct populations P1, P2, and P3. Each of these populations retains the same individuals as P1, with one modified through the addition, removal, or replacement of a stop point. This structure allows for flexible adjustments in the number of stop points, facilitating a thorough exploration of configurations aimed at reducing the UAV’s average energy consumption. However, the approach faces challenges, such as slow convergence due to the generation of three populations per iteration, which results in numerous function evaluations. This also increased memory consumption as the number of stop points grows.

**Fig. 4:**
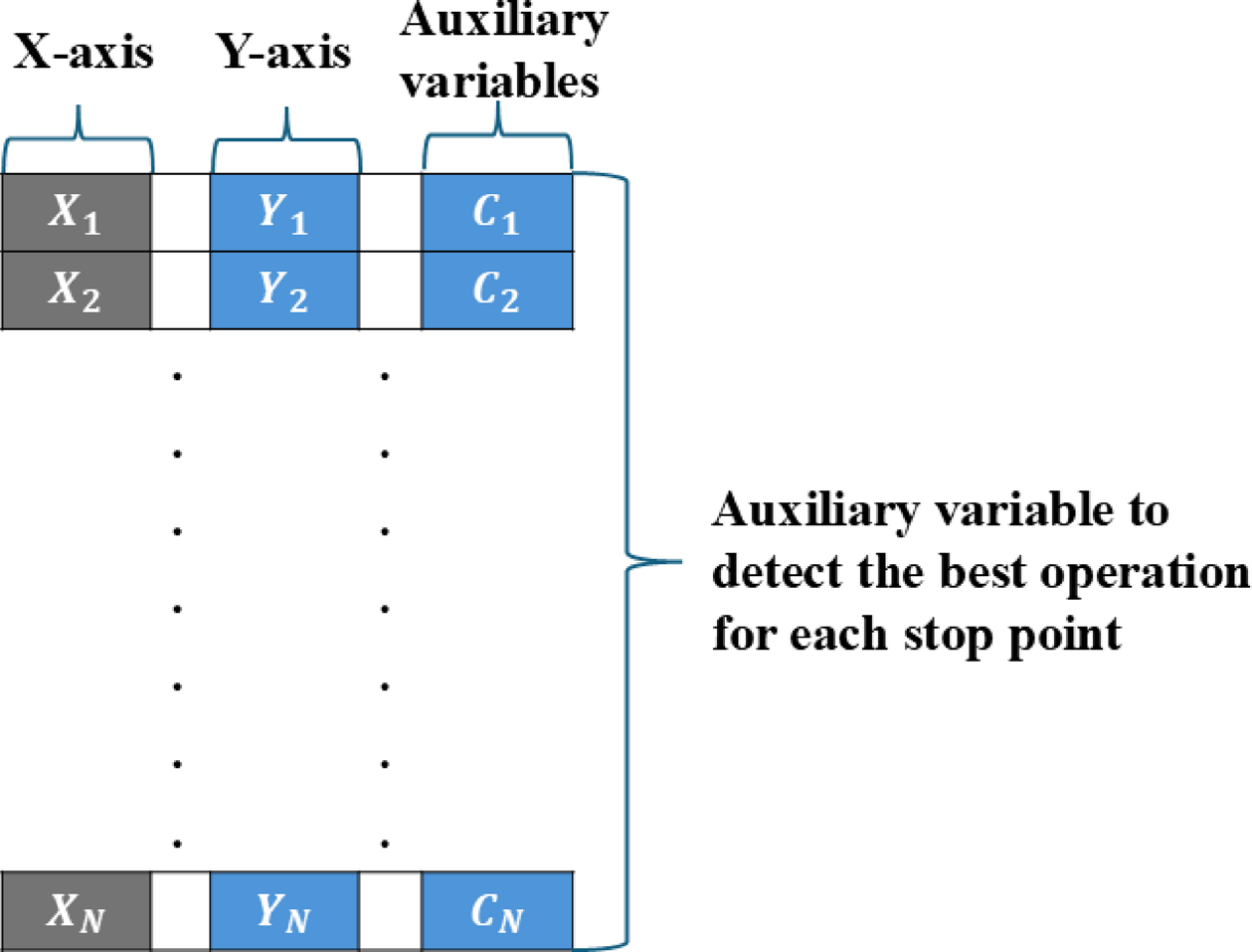
Solution representation for TPO under the oPS mechanism.

**Fig. 5:**
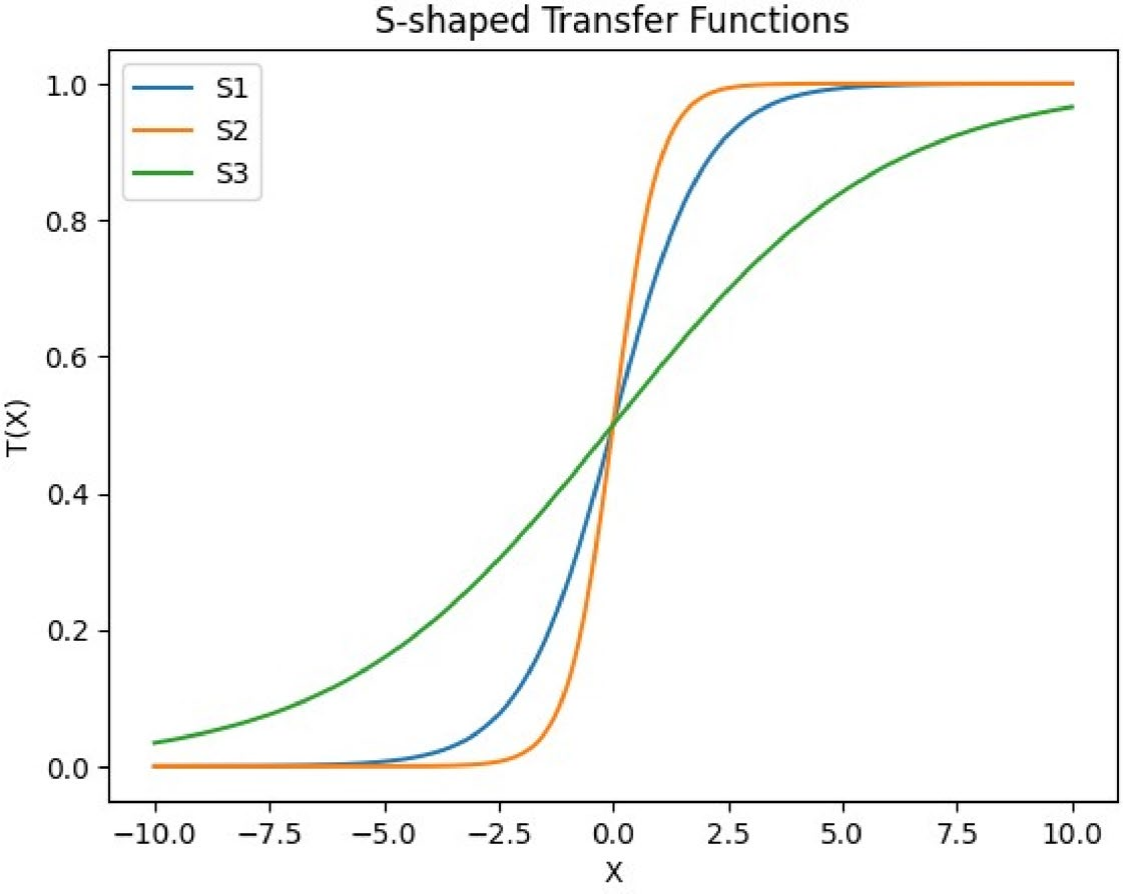
S-shaped transfer function.

**Fig. 6:**
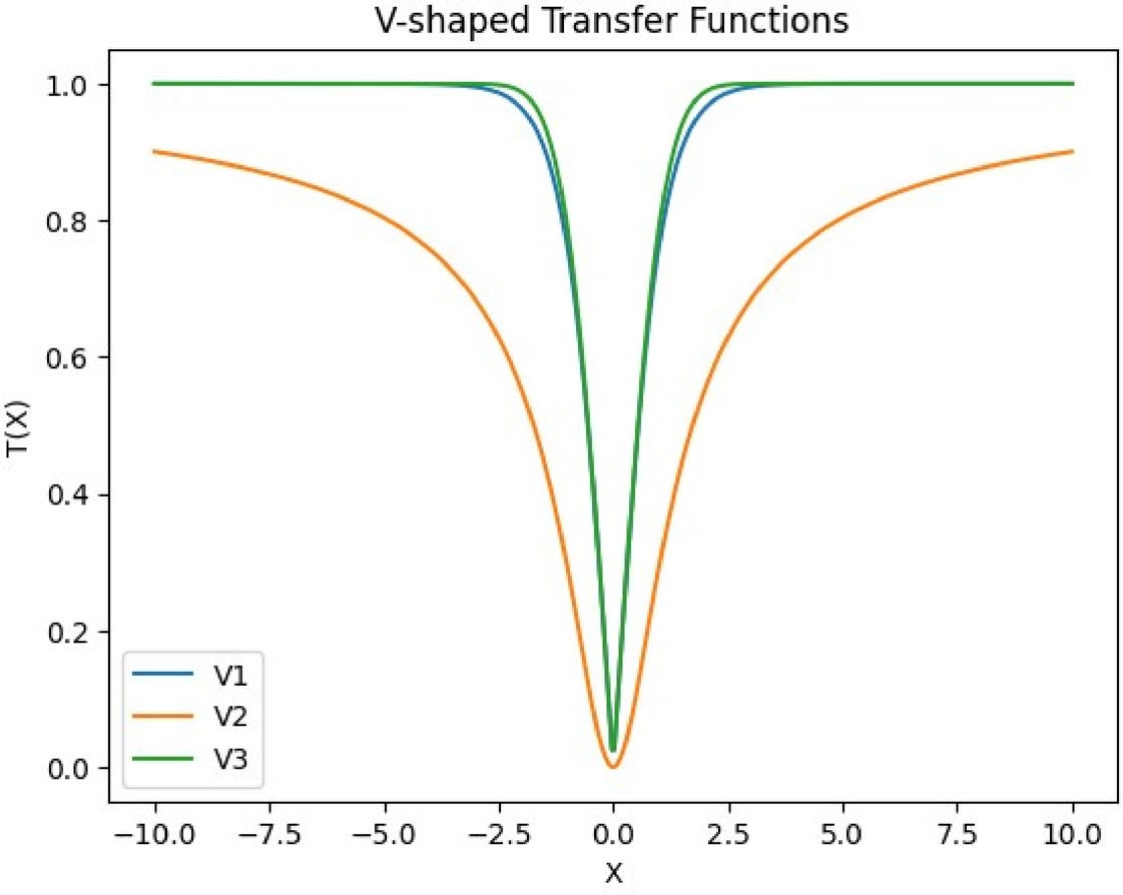
V-shaped transfer function.

To refine the VIPS mechanism further, oPS^85^ was introduced (see Fig. 4). This variant integrates transfer functions to optimize the selection of operations for newly generated stop points, allowing only one operation to be applied per iteration. An auxiliary dimension is added to each population individual to select the optimal operation from the three available options. This dimension is initially initialized randomly based on a normal distribution. Its value is normalized using one of the transfer functions (TF): S-shaped (see Fig. 5) and V-shaped (see Fig. 6), as presented in Table 2 to ensure it falls between 0 and 1. This normalized value is then mapped as follows: 1 represents insertion, −1 represents replacement, and 0 represents deletion, according to the following formula:

32 $$\:{C}_{i}^{a}=\left\{\begin{array}{c}\:1\:\:\:\:\:\:\:\:\:\:\:\:\:\:\:\:if\:{\eta\:}_{1}>\vartheta\:\:\\\:\:0\:\:\:\:\:\:\:\:\:\:\:\:\:\:\:if\:{\eta\:}_{1}>\theta\:\:\\\:-1\:\:\:\:\:\:\:\:\:\:\:\:\:\:\:\:otherwise\end{array}\right.,\forall\:i\:\in\:\left\{1,\:2,\:3,\dots\:,N\right\}$$

Where $\:{\eta\:}_{1}$ is the normalized value, $\:\theta\:\:$and $\:\vartheta\:$ is two predefined thresholding parameters and their values is presented and discussed extensively in the Experiments section.

A whole new population is generated. This population includes all the individuals and stop points that are a part of the current population that were randomly initialized before the optimization process starts. This population is created between the upper and lower limits that have been established for each dimension with respect to m UAVs. Then, FGO is applied to modify the population, creating a new population with a third variable normalized applying just a transfer function among those listed in Table 2. This variable is included in each individual. The values of this variable are then mapped to 1, 0, or −1, according to (32), selecting one of the three operators: insertion, replacement, or removal. The mechanisms of these operators are thoroughly detailed below:

- Insertion: It starts with copying all individuals from P. Then, one individual from the new population $\:{\overrightarrow{x}}_{\:}^{t}\:$is added to this set.
- Replacing : After duplicating P, a randomly chosen member of the duplicated set is substituted with the ith individual from $\:{\overrightarrow{x}}_{\:}^{t}$.
- Removing: Like the others, it begins as a copy of P, but then one individual is randomly removed to modify the population.

The oPS mechanism enhances algorithm performance by randomly selecting an operation from the three available options. This fosters exploration and prevents repeated evaluations of the same operation, improving exploration and exploitation Throughout looking for the optimal procedures for each stop point, according to the formula below:

33 $$\:{C}_{i}^{a}=\left\{\begin{array}{c}rand\left[1,\:0,\:-1\right]\:\:\:\:\:if{\:r}_{1}<\tau\:\\\:\left\{\begin{array}{c}\:1\:\:\:\:\:\:\:\:\:\:\:\:\:\:\:\:if\:{\eta\:}_{1}>\vartheta\:\:\\\:\:0\:\:\:\:\:\:\:\:\:\:\:\:\:\:\:\:if\:{\eta\:}_{1}>\theta\:\:\\\:-1\:\:\:\:\:\:\:\:\:\:\:\:\:\:\:\:otherwise\end{array}\right.\:\:\:\:\:\:\:\:\:\:\:\:\:\:\:\:\:\:\:\:\:\:\:\:\:\end{array},\forall\:i\:\in\:\left\{1,\:2,\:3,\dots\:,N\right\}\:\:\:\right.$$

where $\:{\:r}_{1}$is a randomized variable within the interval [0, 1], and $\:\tau\:$ modulates the operation of the amount of random selection employed in operations and their value is determined based on experimental results. **Algorithm 3** outlines the pseudocode of the oPS mechanism.

**Algorithm 3:**
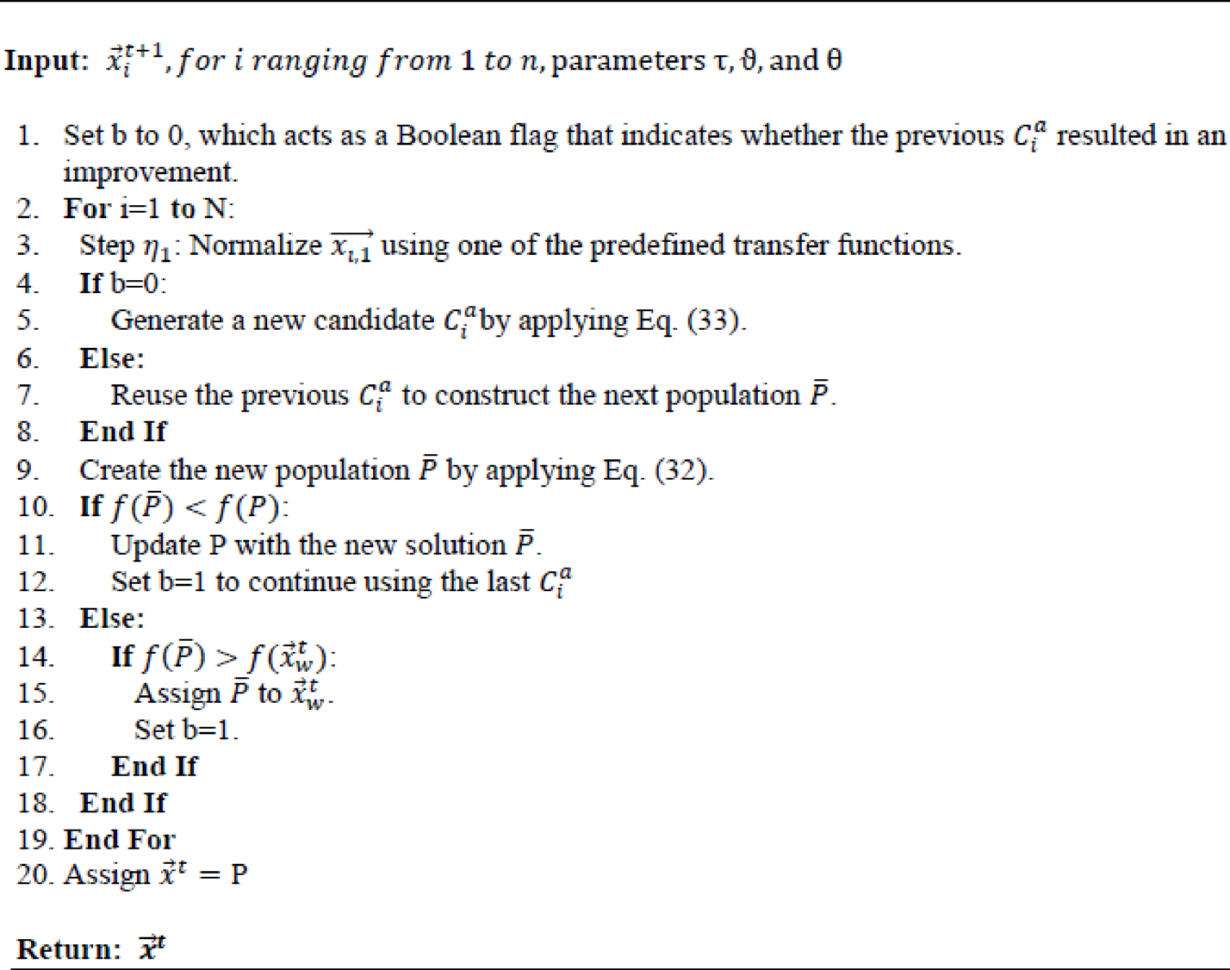
The pseudocode for the oPS mechanism.

Based on the above discussion, this paper adopts oPS as the solution representation mechanism. Unlike Auxiliary Variable-Based and VIPS encoding schemes, oPS maintains a fixed dimensionality while reducing computational and memory overhead. In the proposed framework, oPS is used to represent candidate solutions, whereas the FGO serves as the main search engine to update these solutions.

**Table 2:** TFs with S-shaped and V-shaped.

S-shaped	V-shaped
S1 T$\:\left(\overrightarrow{X}\right)=\frac{1}{1+{e}^{-\overrightarrow{X}}}$	V1 $\:\mathrm{T}\left(\overrightarrow{X}\right)=\left|\mathrm{tanh}\left(\overrightarrow{X}\right)\right|$
S2 $\:\mathrm{T}\left(\overrightarrow{X}\right)=\frac{1}{1+{e}^{-2\overrightarrow{X}}}$	V2 $\:\mathrm{T}\left(\overrightarrow{X}\right)=\left|\frac{a}{\sqrt{1+{\overrightarrow{X}}^{2}}}\right|$
S3 $\:\mathrm{T}\left(\overrightarrow{X}\right)=\frac{1}{1+{e}^{-\overrightarrow{\frac{X}{3}}}}$	V3 $\:\mathrm{T}\left(\overrightarrow{X}\right)=\left|erf\left(\frac{\sqrt{\pi\:}}{2}\overrightarrow{X}\right)\right|$

### Gaussian mutation

GM^84^ is integrated into the proposed FGO algorithm as a stochastic perturbation technique to improve population variety and alleviate premature convergence. GM introduces changes to the existing solution by adding stochastic values generated according to a Gaussian distribution defined by its mean and standard deviation. The mutation is controlled by a mutation rate γ and mutation strength δ and their value is determined based on experimental results. Increasing the mutation rate increases the potential that a mutation will take place, while mutation strength affects the changes that are applied to the variables forming the solution. The normal distribution is denoted by the notation $\:N\left(\mu,\sigma\right)$, with µ representing the mean and σ representing the standard deviation of the distribution. Mathematically, Gaussian mutation can be expressed as:

34 $$\:{X}_{i,j}=\:\left\{\begin{array}{c}{X}_{i,j}+N\left(\mu,\sigma\right)\times\delta\:,\:\:\:\:\:\:\:if\:rand\:\left(\mathrm{0,1}\right)\:<\gamma\:\:\\\:\:{X}_{i,j,}\:\:otherwise\:\:\:\:\:\:\:\:\:\:\:\:\:\:\:\:\:\:\:\:\:\:\:\:\:\:\:\:\:\:\:\:\:\:\:\:\:\:\:\:\:\:\:\:\:\:\:\:\:\:\:\:\:\:\:\end{array}\right.$$

Within the FGO optimization cycle, GM is applied after each population update, introducing localized perturbations around fungal growth solutions. This integration complements global and local search mechanisms of FGO by preserving population diversity and facilitating escape from local optima while steering the search process to achieve faster convergence as detailed in Algorithm 4.

**Algorithm 4:**
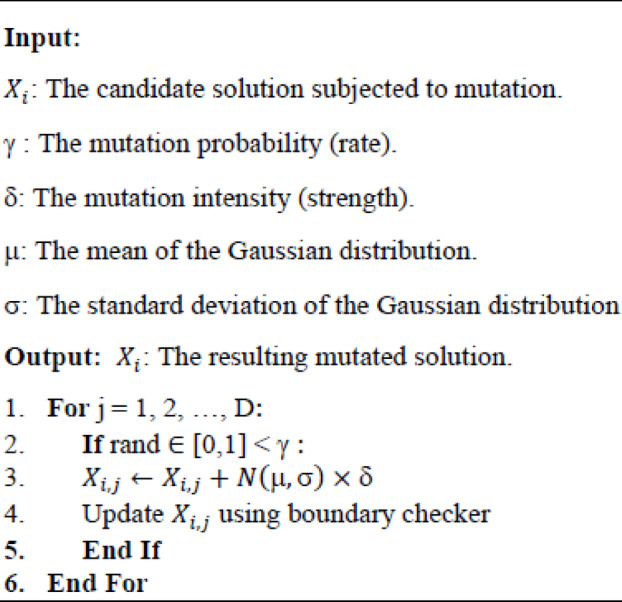
The pseudocode for Gaussian mutation procedure.

### Local exploitation using differential evolution and low-complexity greedy

In the proposed algorithm, the exploitation phase integrates DE and an LCG strategy to refine candidate solutions and construct efficient UAV trajectories. At each iteration, DE is applied immediately after generating the population solutions within the FGO algorithm. For each individual solution $\:{x}_{i}$, DE generates a trial vector through mutation and crossover operations involving randomly selected individuals from the population. These operations introduce localized perturbations to the decision variables associated with UAV deployment and resource allocation, thereby enabling an effective local search around the current solutions. Subsequently, the fitness values of both trial and original solutions are evaluated to guide the selection process, where the solution with superior fitness is retained for further processing.

Following the DE-based refinement, K-means clustering is applied to organize UAVs that are spatially close into coherent clusters, enabling the design of structured trajectories. This clustering step does not alter the optimized deployment variables; instead, it simplifies the trajectory planning process and reduces potential path overlaps.

After assigning stop points to the $\:m$UAVs, it becomes necessary to determine an appropriate visiting sequence for these points to ensure economically efficient operation. To this end, the LCG algorithm, originally introduced in^78^, is applied to construct each UAV trajectory. The starting node is determined by identifying the stop point with the minimum distance to the centroid of the assigned stop points. Subsequently, the nearest neighboring stop point is iteratively added based on distance minimization until all assigned points are included, as detailed in Algorithm 5.

Within the proposed framework, LCG is utilized at two critical stages. Initially, it is applied after population initialization to generate a feasible visiting sequence that guides the early search process of FGO. Later, after FGO and DE optimized the allocation of stop points to UAVs, LCG is reapplied to construct the final trajectories with reduced computational complexity. This tight integration of FGO’s global exploration capability, DE’s local refinement mechanism, and LCG’s efficient sequencing strategy accelerates convergence and enhances the overall solution quality.

**Algorithm 5:**
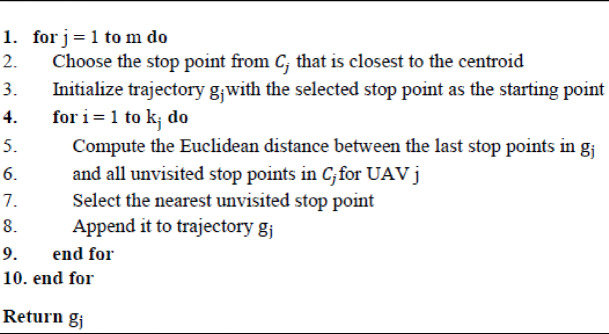
The pseudocode for the LCG.

### Pseudocode for FGODE

The proposed FGODE algorithm integrates multiple optimization mechanisms to achieve a balanced exploration–exploitation process for UAV trajectory planning. FGO serves as the main search engine that explores the search space effectively. The DE algorithm is applied selectively during the exploitation phases to refine promising solutions, enhancing local search accuracy.

**Fig. 7:**
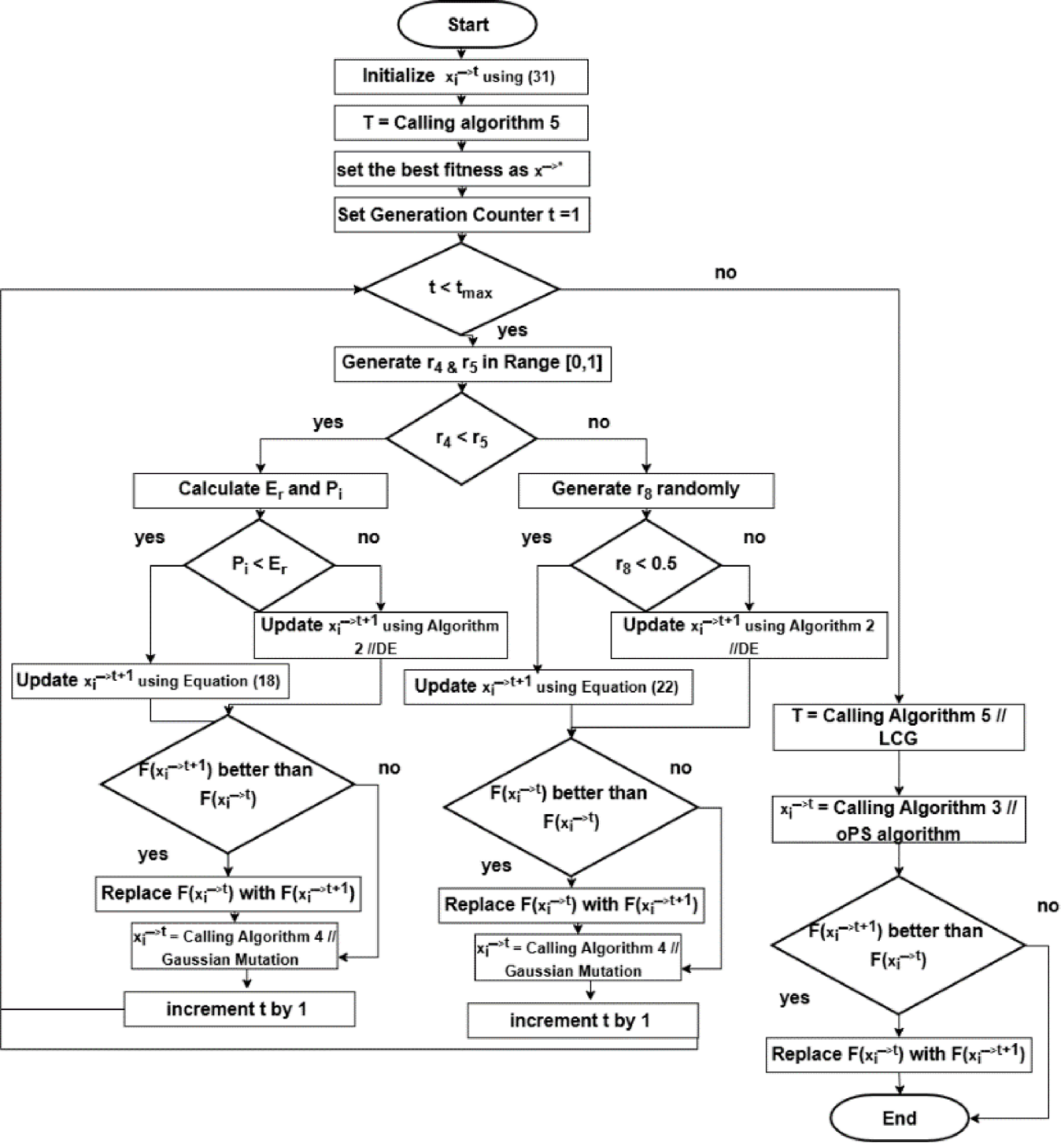
Flowchart of the FGODE algorithm.

EOBL is applied once at initialization to generate diverse starting solutions, while the Gaussian Mutation operator is employed iteratively to maintain population diversity while avoiding early convergence. The LCG algorithm is used to construct efficient visiting sequences for the stop points both after initialization and at the end of each iteration, ensuring computationally efficient UAV trajectories.

Additionally, the oPS mechanism is applied at each iteration to update solutions based on optimal operation selection. Together, these components form a hybrid framework where FGO drives global exploration, DE improves local refinement, LCG organizes trajectories, elite opposition enhances diversity at the start, GM maintains stochastic variability, and oPS ensures effective operation application, as illustrated in Fig. 7.

## Settings for experiments

The effectiveness of the suggested FGODE algorithm in trajectory planning for MEC systems utilizing multiple UAVs is assessed in this work. The experiments consider eight deployment instances, where the number of IoT devices (m) varies as {60, 80, 100, 120, 140, 160, 180, 200}. These devices are placed at random throughout a 1000-meter by 1000-meter area, while 4 UAVs hover 200 m above ground. The data sizes $\:{M}_{i},\forall\:i\in\:\left\{\mathrm{1,2},\dots\:,m\right\}\:$produced at random within the range [1, 10³] MB. The parameter δ is initially set to 5, and different values are later used to evaluate the proposed algorithm’s capability in minimizing energy consumption under various conditions. The transmission power $\:{P}_{i},\forall\:i\in\:\left\{\mathrm{1,2},\dots\:,m\right\}\:$is fixed at 0.1 W. The parameters $\:{G}_{0}$ and $\:{\beta\:}_{2}\:$are assigned values of 30 dB and 250 dBm, respectively. The system bandwidth $\:B\:$is set to 1 MHz, while the UAV computing power $\:{p}_{u}\:$and control parameter $\in$are set to 1000 W and 10,000, respectively. Series of experiments was carried out to assess how effective the proposed FGODE algorithm in comparison with seven competitive optimizers, namely Puma optimizer (PO)^86^, Narwhal Optimization (NWO)^87^, Eel and grouper optimizer (EGO)^88^, Quadratic Interpolation Optimization (QIO)^89^, and Water Optimization Algorithm)WOA)^90^. Furthermore, two hybrid optimization algorithms namely Hybrid Genetic Algorithm and Sperm Swarm Optimization (HGASSO)^91^ and Hybrid Sperm Swarm Optimization and Gravitational Search Algorithm (HSSOGSA)^92^. During these experiments, all optimizers were run separately for 25 independent trials, with the same population size which is set equal to the number of IoT devices in each instance and remains fixed across all independent runs, maximum number of iterations, and termination conditions applied to ensure a fair and consistent comparison. Followed by an analysis of the obtained fitness values using multiple performance metrics to ensure a comprehensive comparison. Among these metrics is the p-value (PV) obtained from the Wilcoxon rank-sum (WRS) test, the average energy consumption (AEC), the standard deviation (SD) to evaluate how consistently each algorithm performs, and the Friedman mean rank (FK) to assess relative ranking performance among all algorithms. The PV metric indicates the statistical significance of the proposed algorithm versus the competing methods, AEC reflects the overall quality of solutions, SD measures the robustness of each optimizer across different runs, and FK provides a comparative ranking among all algorithms. To ensure a fair evaluation, all competing optimization control settings were set based on their respective references, while the maximum allowed iterations (Tₘₐₓ) was uniformly fixed at 10,000 for all algorithms. All the experiments in this work are executed using MATLAB R2021a. The device on which it is installed has a 2.80 GHz Intel^®^ Core™ i7 processor and 16 GB memory, a 4 GB graphics card, and 1.14 TB of storage. The laptop operates on a Windows 10 Pro, 64-bit edition system.

## Findings and discussions

An evaluation of the performance of FGODE algorithm across several configurations is presented in this section. Initially, it estimated the optimal values for the algorithm’s controlling parameter. Secondly, it examines how effective the different transfer functions. Next, the hybrid optimizer FGODE is compared against several recently developed metaheuristic algorithms to highlight its efficiency and robustness in solving UAV-assisted MEC deployment problems.

### Parameter tuning

**Fig. 8:**
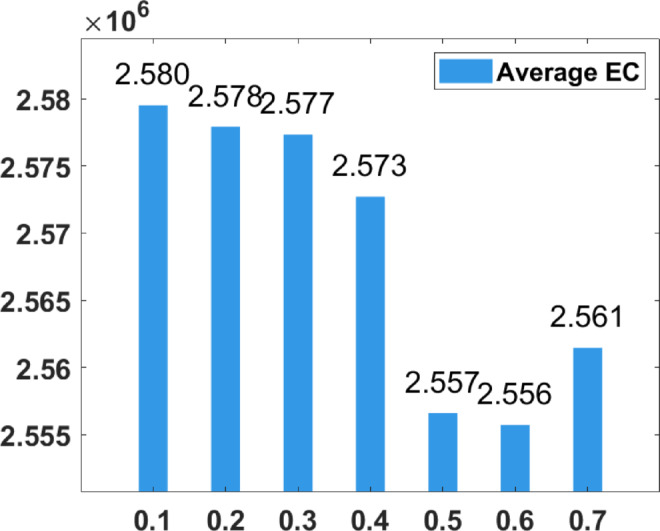
EC values averaged per parameter setting to determine θ.

**Fig. 9:**
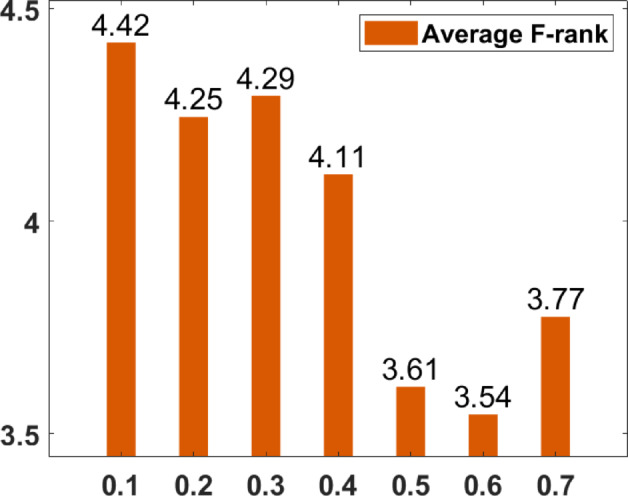
F-rank values averaged per parameter setting to determine θ.

**Fig. 10:**
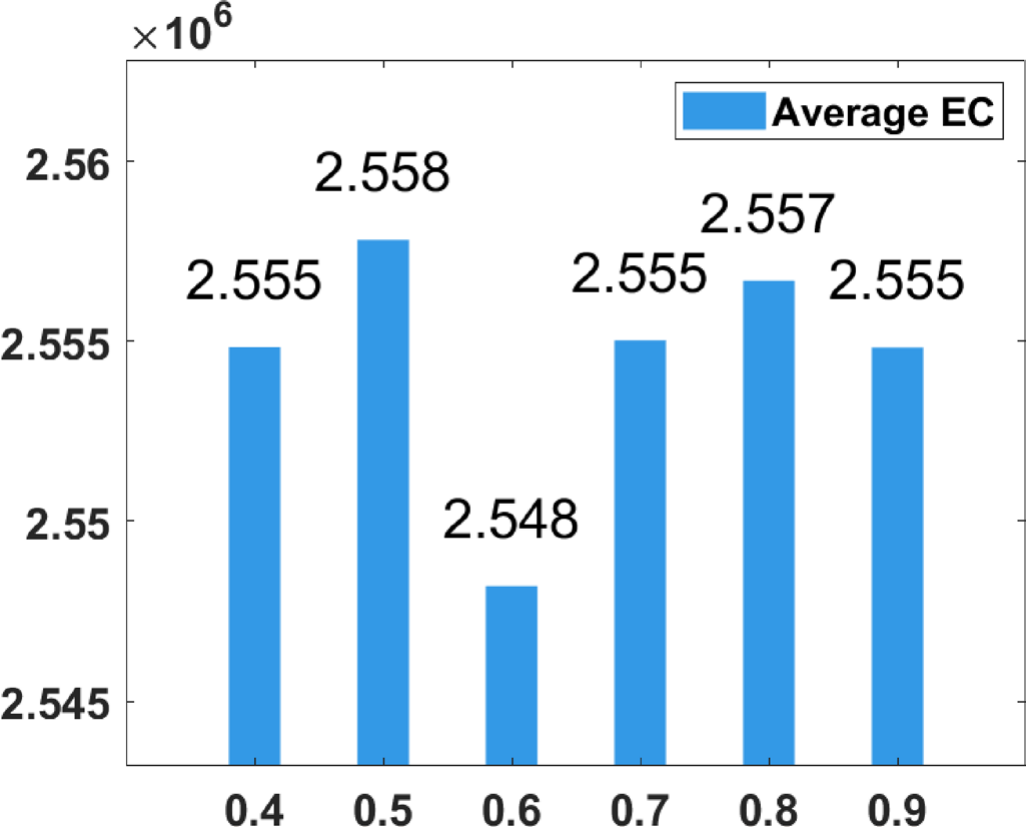
EC values averaged per parameter setting to determine ϑ.

**Fig. 11:**
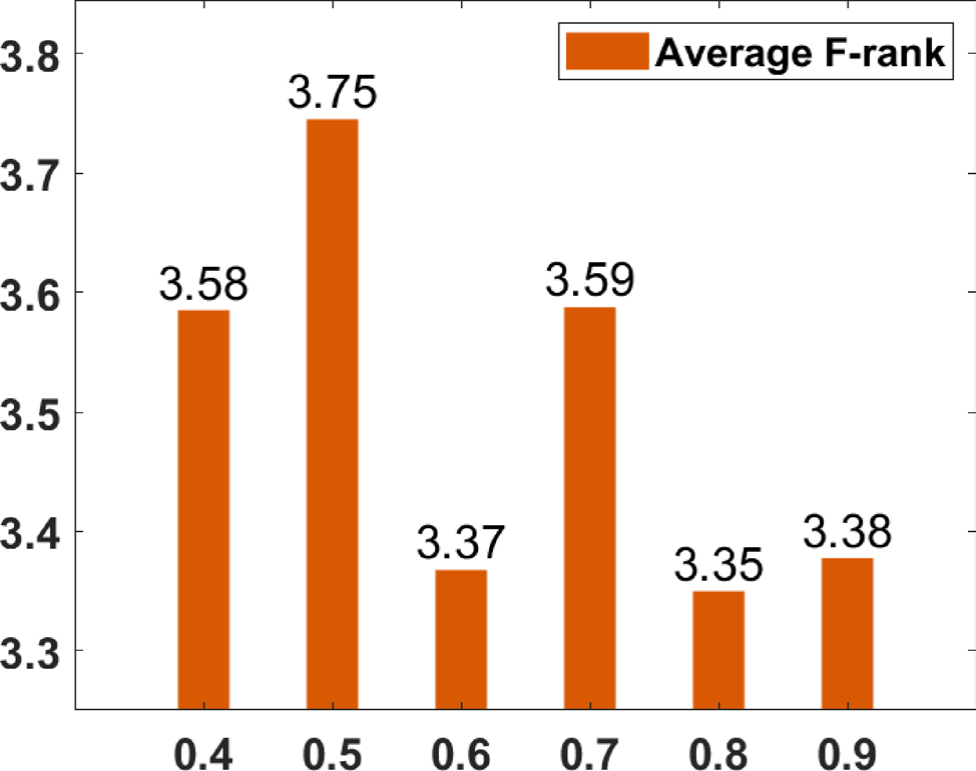
F-rank values averaged per parameter setting to determine ϑ.

**Fig. 12:**
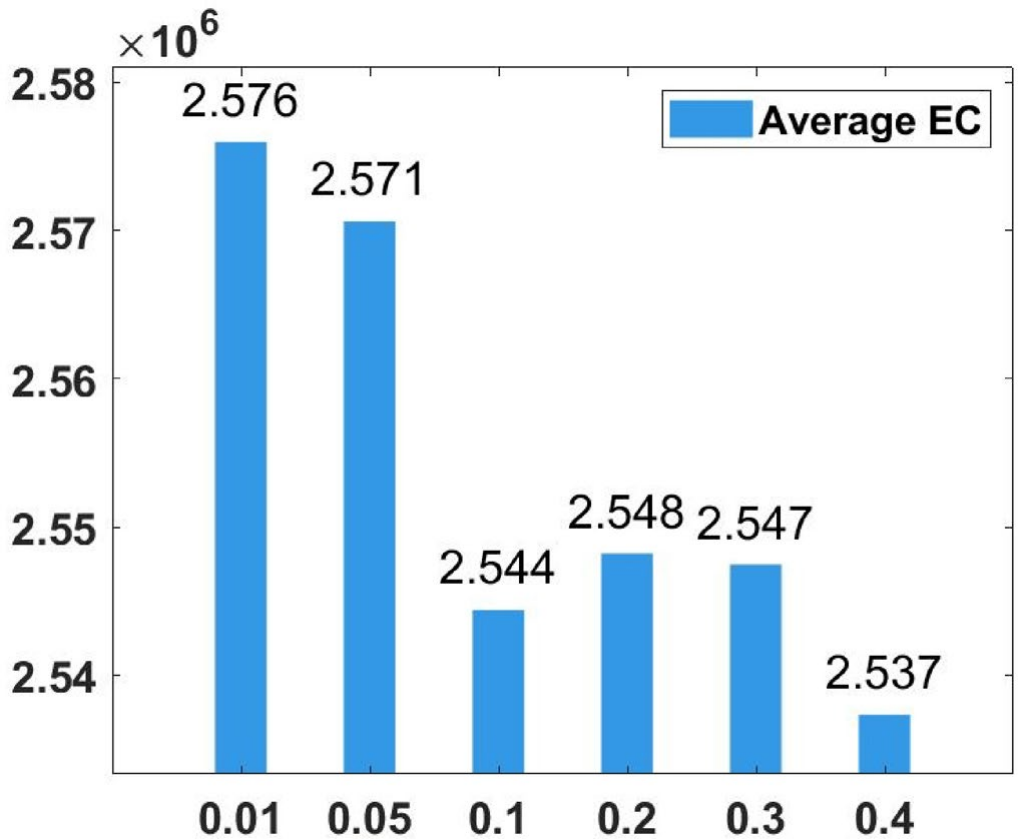
EC values averaged per parameter setting to determine τ.

**Fig. 13:**
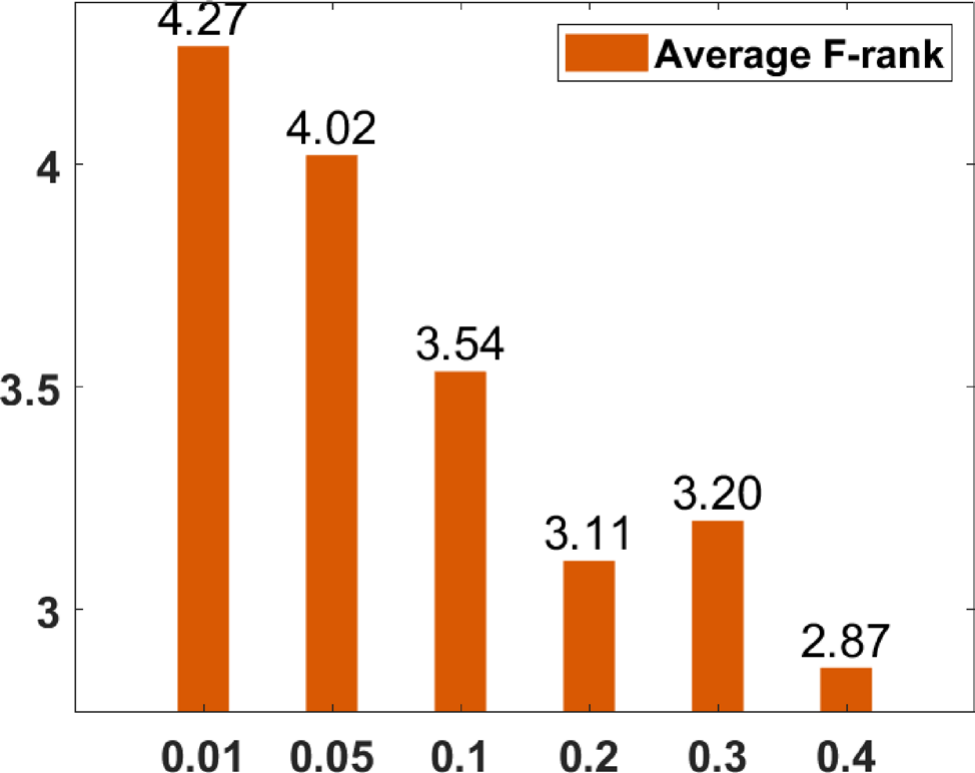
F-rank values averaged per parameter setting to determine τ.

**Fig. 14:**
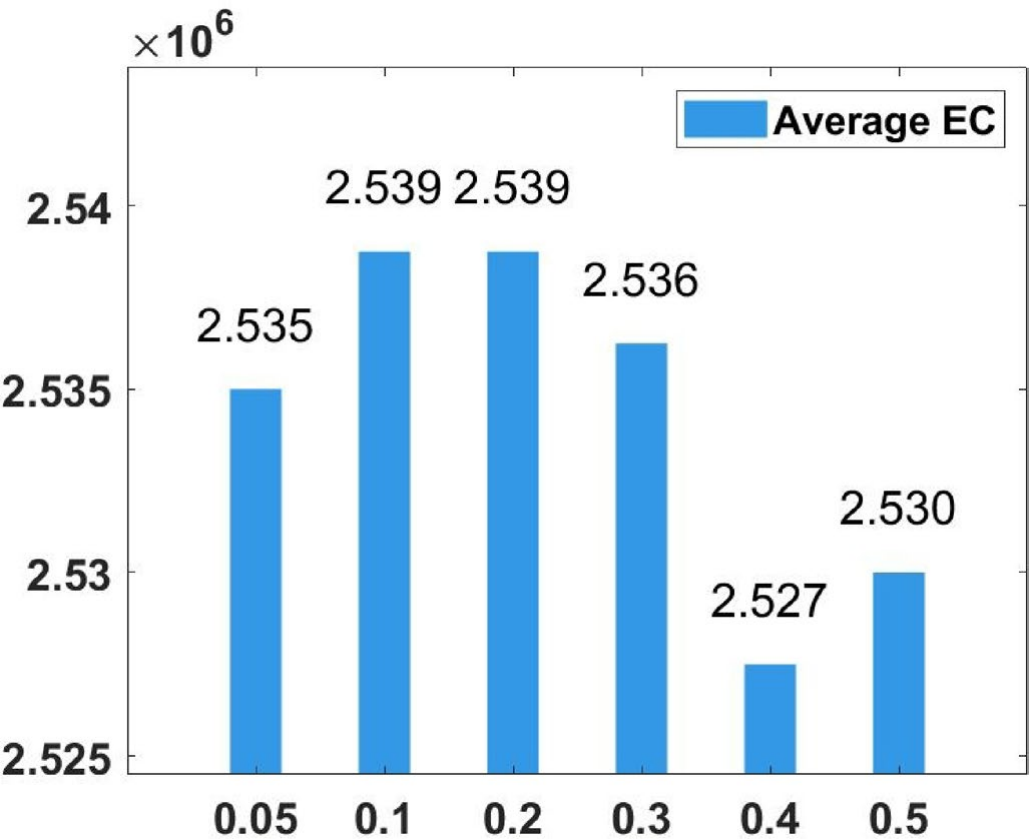
EC values averaged per parameter setting to determine $\gamma$.

**Fig. 15:**
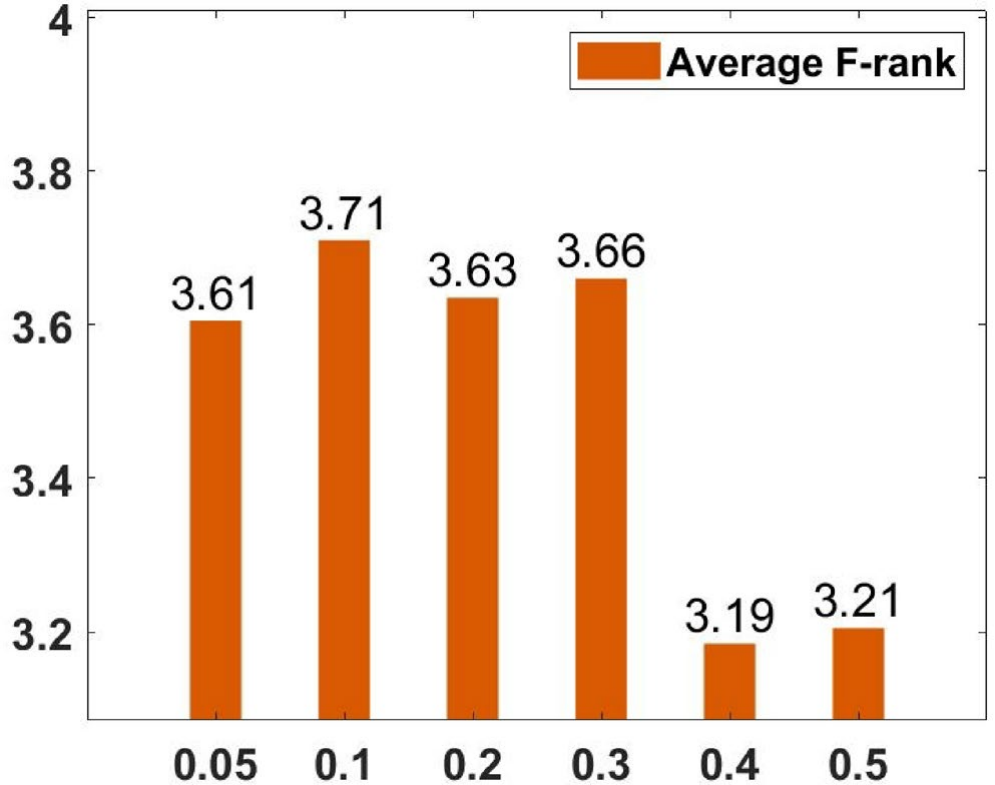
F-rank values averaged per parameter setting to determine $\gamma$.

**Fig. 16:**
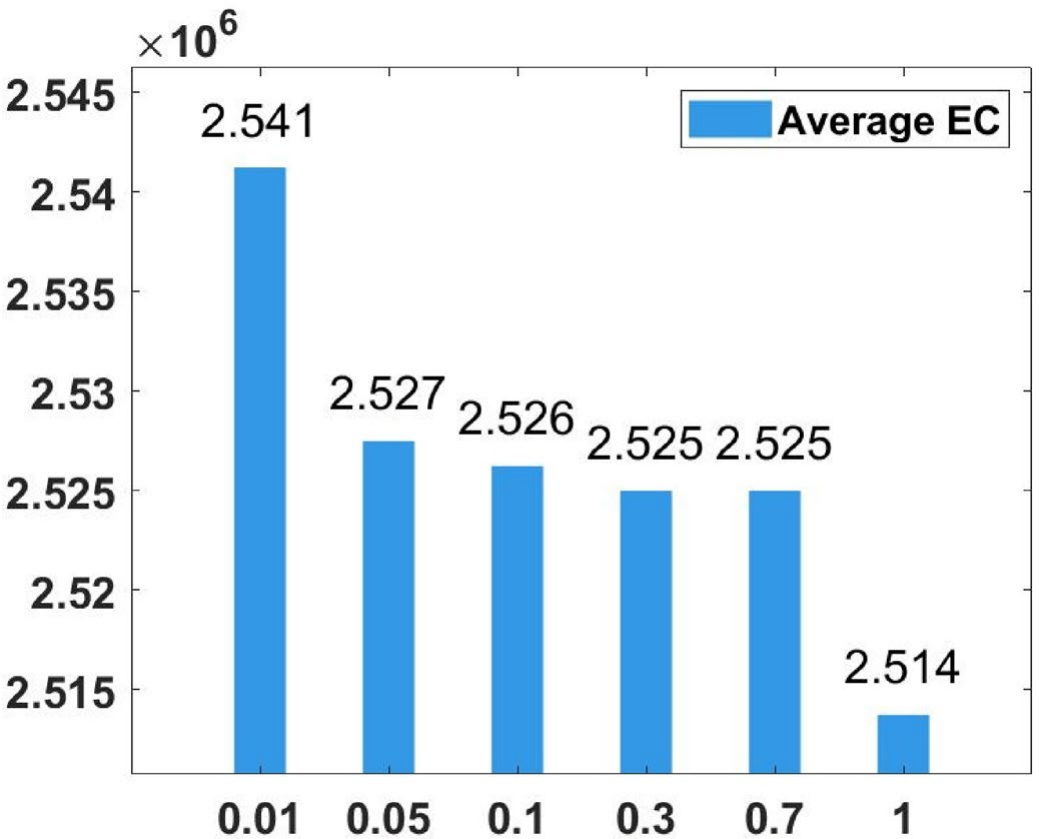
EC values averaged per parameter setting to determine δ.

**Fig. 17:**
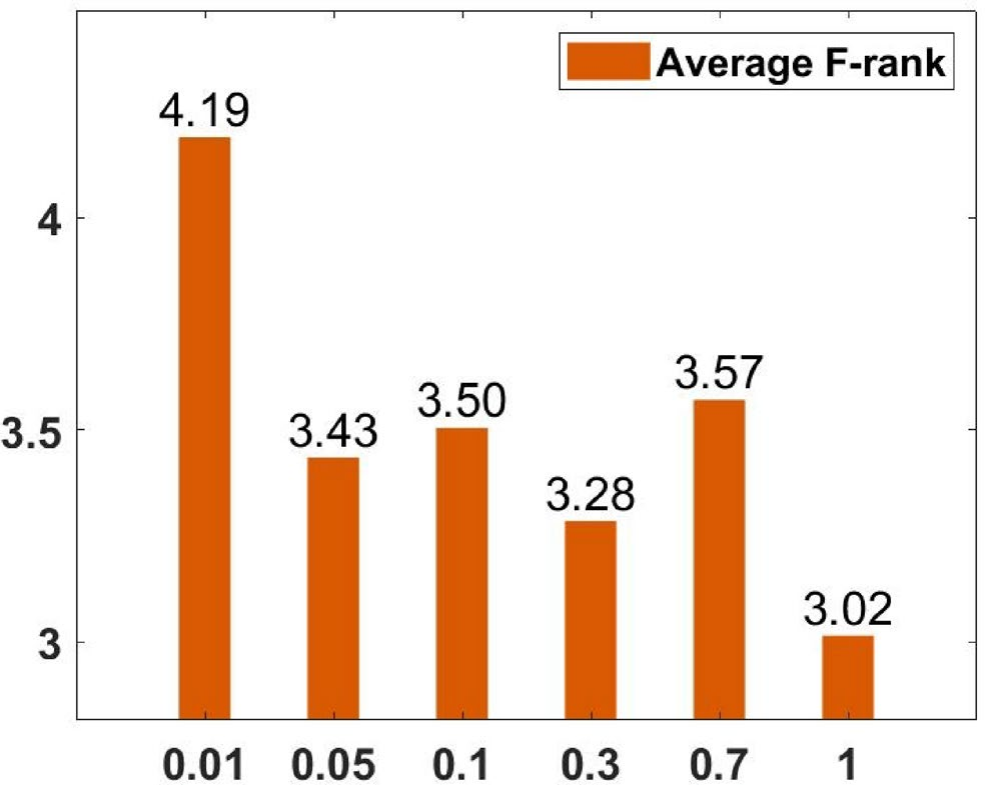
F-rank values averaged per parameter setting to determine δ.

In the proposed FGODE algorithm, the encoding mechanism includes three key parameters that must be carefully tuned to enhance its overall optimization performance when integrated with the FGODE hybrid algorithm. To identify the most suitable settings, the study involved performing multiple experiments by varying each parameter’s value within specific ranges. For instance, the first parameter, θ, which controls the interplay of exploration versus exploitation during encoding stage, was tested under multiple values ranging from 0.1 to 0.7. Experimental outcomes, summarized in Table 3, and shown in Fig. 8, and Fig. 9, reveal that setting θ = 0.6 yields the most stable and energy-efficient outcomes based upon AEC and FK, as it provides a balanced probability between generating new UAV stop points and retaining promising ones. Similarly, the second parameter, ϑ, which influences the adaptive probability of replacement operation within the encoding stage, was tested under multiple values ranging from 0.4 to 0.9. As determined by the results of AEC and FK reported in Table 4, and shown through illustrations in Fig. 10, and Fig. 11, ϑ = 0.6 was selected as the optimal value, as it effectively maintains a suitable trade-off between preserving beneficial positions and discarding redundant ones, minimizing unnecessary UAV movements and total energy cost. The third parameter, τ, which determines probability of randomization to reinforce the algorithm’s exploration efficiency, was fine-tuned through multiple experiments using values in the range [0.01, 0.4]. As shown in Table 5, and shown through visual representations in Fig. 12, and Fig. 13, the best performance according to AEC and FK was obtained when τ = 0.4, since this setting introduces enough stochastic behavior to examine unvisited sections of the solution space without destabilizing convergence. Furthermore, the Gaussian mutation includes two additional parameters tuned experimentally: the mutation rate γ and the mutation strength δ, which control the probability and magnitude of the applied perturbations, respectively. The mutation rate γ was tested using values from 0.05 to 0.5, and as shown in Table 6, and shown through visual representations in Fig. 14, and Fig. 15, $\:=0.4$ achieved an optimal trade-off between exploration and convergence stability. Similarly, the mutation strength $\:\delta\:$ was tested using the values 0.01, 0.05, 0.1, 0.3, 0.7 and 1. As shown in Table 7, and illustrated in Fig. 16, and Fig. 17, the optimal performance was obtained at $\:\delta\:=1$, providing sufficient diversification without degrading convergence. These optimized parameter values (θ = 0.6, ϑ = 0.6, τ = 0.4, γ = 0.4 and δ = 1) were thus adopted in all subsequent simulations to ensure consistent and effective behavior of the proposed hybrid FGODE algorithm.

**Table 3:** Adjusting the parameter θ.

		0.1	0.2	0.3	0.4	0.5	0.6	0.7
60	AEC	1.09E + 06	1.09E + 06	1.09E + 06	1.09E + 06	**1.09E + 06**	**1.09E + 06**	1.10E + 06
	F-rank	4.16	3.68	4.28	4	**3.5**	**3.5**	4.88
80	AEC	**1.59E + 06**	1.61E + 06	1.61E + 06	1.60E + 06	1.59E + 06	1.59E + 06	1.59E + 06
	F-rank	**3.64**	4.12	4.48	4	3.92	3.92	3.92
100	AEC	2.05E + 06	2.06E + 06	2.05E + 06	2.04E + 06	**2.04E + 06**	**2.04E + 06**	2.04E + 06
	F-rank	4.08	4.4	**3.44**	3.48	4.18	4.18	4.24
120	AEC	2.18E + 06	2.19E + 06	2.16E + 06	2.17E + 06	2.17E + 06	2.17E + 06	**2.15E + 06**
	F-rank	4.32	4.56	3.84	4	4.12	4.2	**2.96**
140	AEC	2.83E + 06	2.83E + 06	2.82E + 06	2.82E + 06	2.78E + 06	**2.78E + 06**	2.80E + 06
	F-rank	4.6	4.48	4.8	4.44	3.4	**2.88**	3.4
160	AEC	3.24E + 06	3.21E + 06	3.21E + 06	**3.18E + 06**	3.19E + 06	**3.18E + 06**	3.21E + 06
	F-rank	4.92	4.36	4.2	3.6	3.72	**3.4**	3.8
180	AEC	3.63E + 06	3.62E + 06	3.65E + 06	3.64E + 06	3.59E + 06	**3.59E + 06**	3.60E + 06
	F-rank	4.88	4.28	5	4.4	3.16	**2.8**	3.48
200	AEC	4.04E + 06	4.02E + 06	4.03E + 06	4.04E + 06	**3.98E + 06**	4.00E + 06	4.00E + 06
	F-rank	4.76	4.08	4.32	4.96	**2.88**	3.48	3.52

Values in **bold** indicate the optimal results.

**Table 4:** Adjusting the parameter ϑ.

		0.4	0.5	0.6	0.7	0.8	0.9
60	AEC	1.10E + 06	1.10E + 06	**1.09E + 06**	**1.09E + 06**	**1.09E + 06**	**1.09E + 06**
	F-rank	4.44	4.32	**3.06**	**3.06**	**3.06**	**3.06**
80	AEC	1.59E + 06	1.60E + 06	**1.59E + 06**	1.60E + 06	1.59E + 06	1.61E + 06
	F-rank	3.48	3.68	**3.16**	3.64	3.36	3.68
100	AEC	2.04E + 06	2.05E + 06	2.04E + 06	2.06E + 06	2.04E + 06	**2.03E + 06**
	F-rank	3.2	3.72	3.6	4.16	**3.12**	3.2
120	AEC	2.17E + 06	**2.16E + 06**	2.16E + 06	2.16E + 06	2.16E + 06	2.19E + 06
	F-rank	3.6	**3.24**	3.44	3.32	3.44	3.96
140	AEC	2.78E + 06	2.79E + 06	**2.76E + 06**	2.79E + 06	2.79E + 06	2.79E + 06
	F-rank	3.48	3.96	**2.76**	3.72	3.48	3.6
160	AEC	3.17E + 06	3.17E + 06	3.19E + 06	3.18E + 06	3.17E + 06	**3.14E + 06**
	F-rank	3.48	3.68	4.12	3.64	3.48	**2.6**
180	AEC	3.60E + 06	3.60E + 06	3.58E + 06	**3.58E + 06**	3.62E + 06	3.62E + 06
	F-rank	3.72	3.64	3.36	3.44	**3.24**	3.6
200	AEC	3.98E + 06	3.99E + 06	**3.97E + 06**	3.98E + 06	3.99E + 06	3.98E + 06
	F-rank	**3.28**	3.72	3.44	3.72	3.52	3.32

Values in **bold** indicate the optimal results.

**Table 5:** Adjusting the parameter τ.

		0.01	0.05	0.1	0.2	0.3	0.4
60	AEC	1.12E + 06	1.11E + 06	1.10E + 06	1.09E + 06	1.10E + 06	**1.08E + 06**
	F-rank	4.68	3.96	3.64	2.8	3.56	**2.36**
80	AEC	1.61E + 06	1.61E + 06	1.60E + 06	1.59E + 06	1.58E + 06	**1.57E + 06**
	F-rank	4.4	4.4	3.76	3.12	3	**2.32**
100	AEC	2.08E + 06	2.06E + 06	2.05E + 06	**2.04E + 06**	2.05E + 06	2.04E + 06
	F-rank	4.64	3.72	3.28	**2.88**	3.36	3.12
120	AEC	2.18E + 06	2.19E + 06	**2.05E + 06**	2.16E + 06	2.15E + 06	2.15E + 06
	F-rank	4.28	4.24	3.24	3.36	2.96	**2.92**
140	AEC	2.80E + 06	2.82E + 06	2.78E + 06	**2.76E + 06**	2.78E + 06	2.7794 + 06
	F-rank	3.88	4.44	3.24	**2.76**	3.28	3.4
160	AEC	3.20E + 06	3.18E + 06	3.18E + 06	3.19E + 06	3.16E + 06	**3.15E + 06**
	F-rank	4.12	3.6	3.36	3.8	3.12	**3**
180	AEC	3.60E + 06	3.62E + 06	3.59E + 06	3.58E + 06	3.57E + 06	**3.57E + 06**
	F-rank	3.92	4.08	3.76	3.12	3.08	**3.04**
200	AEC	4.02E + 06	3.99E + 06	4.01E + 06	3.97E + 06	3.98E + 06	**3.96E + 06**
	F-rank	4.2	3.72	4	3.04	3.24	**2.8**

Values in **bold** indicate the optimal results.

**Table 6:** Adjusting the parameter $\gamma$.

		0.05	0.1	0.2	0.3	0.4	0.5
60	AEC	1.06E + 06	1.06E + 06	1.06E + 06	1.06E + 06	**1.06E + 06**	1.06E + 06
	F-rank	3.68	3.4	3.8	3.48	**3.2**	3.44
80	AEC	1.55E + 06	1.56E + 06	1.56E + 06	1.55E + 06	1.55E + 06	**1.54E + 06**
	F-rank	3.2	4.12	3.68	3.72	3.4	**2.88**
100	AEC	2.00E + 06	2.01E + 06	1.99E + 06	2.00E + 06	**1.98E + 06**	2.02E + 06
	F-rank	3.92	3.72	3.52	3.8	**2.92**	3.12
120	AEC	2.14E + 06	2.13E + 06	2.13E + 06	2.14E + 06	**2.11E + 06**	2.12E + 06
	F-rank	3.84	3.52	3.64	3.92	**2.96**	3.12
140	AEC	**2.76E + 06**	2.78E + 06	2.78E + 06	2.77E + 06	2.77E + 06	2.77E + 06
	F-rank	**2.92**	3.76	3.64	3.84	3.32	3.52
160	AEC	**3.15E + 06**	3.17E + 06	3.18E + 06	3.16E + 06	3.18E + 06	3.16E + 06
	F-rank	3.56	3.84	3.64	3.36	3.36	**3.24**
180	AEC	3.62E + 06	3.60E + 06	3.61E + 06	3.61E + 06	3.59E + 06	**3.58E + 06**
	F-rank	4.12	3.64	3.76	3.48	3.16	**2.84**
200	AEC	4.00E + 06	4.00E + 06	4.00E + 06	4.00E + 06	**3.98E + 06**	3.99E + 06
	F-rank	3.6	3.68	3.4	3.68	**3.16**	3.48

Values in **bold** indicate the optimal results.

**Table 7:** Adjusting the parameter δ.

m		0.01	0.05	0.1	0.3	0.7	1
60	AEC	1.06E + 06	1.06E + 06	1.05E + 06	1.05E + 06	1.05E + 06	**1.04E + 06**
	F-rank	3.84	3.8	3.56	3.12	4	**2.68**
80	AEC	1.57E + 06	1.55E + 06	1.55E + 06	1.55E + 06	**1.54E + 06**	1.55E + 06
	F-rank	4.36	3.28	**3.16**	3.52	3.28	3.4
100	AEC	2.01E + 06	1.98E + 06	**1.98E + 06**	1.98E + 06	2.00E + 06	1.99E + 06
	F-rank	4.56	3.32	**3.16**	2.92	3.84	3.2
120	AEC	2.15E + 06	**2.11E + 06**	2.13E + 06	2.12E + 06	2.12E + 06	2.12E + 06
	F-rank	4.6	**2.84**	3.52	3.28	3.32	3.44
140	AEC	2.76E + 06	2.77E + 06	2.77E + 06	2.78E + 06	2.76E + 06	**2.73E + 06**
	F-rank	3.96	3.84	3.68	3.68	3.48	**2.36**
160	AEC	3.19E + 06	3.18E + 06	3.16E + 06	3.16E + 06	3.14E + 06	**3.12E + 06**
	F-rank	4.44	3.76	3.84	3.2	3.36	**2.4**
180	AEC	3.60E + 06	3.59E + 06	3.59E + 06	3.58E + 06	3.60E + 06	**3.58E + 06**
	F-rank	3.96	3.48	3.56	3.16	3.72	**3.12**
200	AEC	3.99E + 06	**3.98E + 06**	3.98E + 06	3.98E + 06	3.99E + 06	3.98E + 06
	F-rank	3.8	**3.16**	3.56	3.4	3.56	3.52

Values in **bold** indicate the optimal results.

### Impact of employing multiple transfer functions

**Fig. 18:**
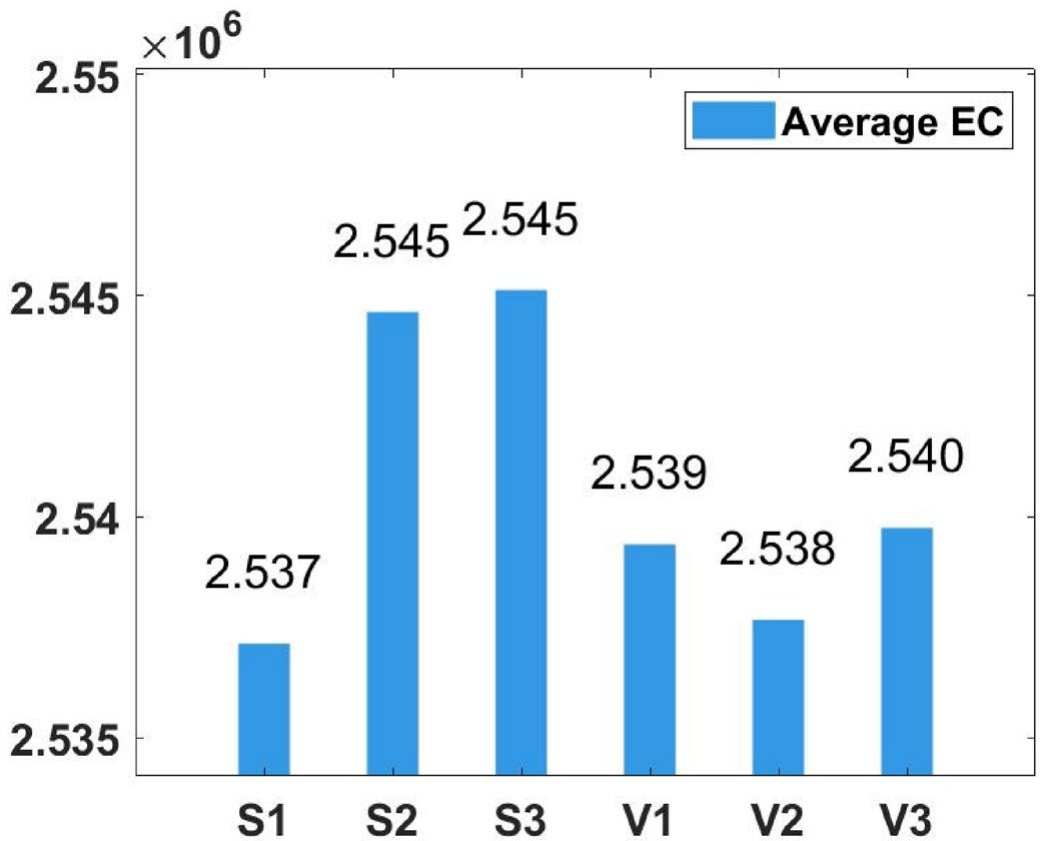
Average EC values for each TF.

**Fig. 19:**
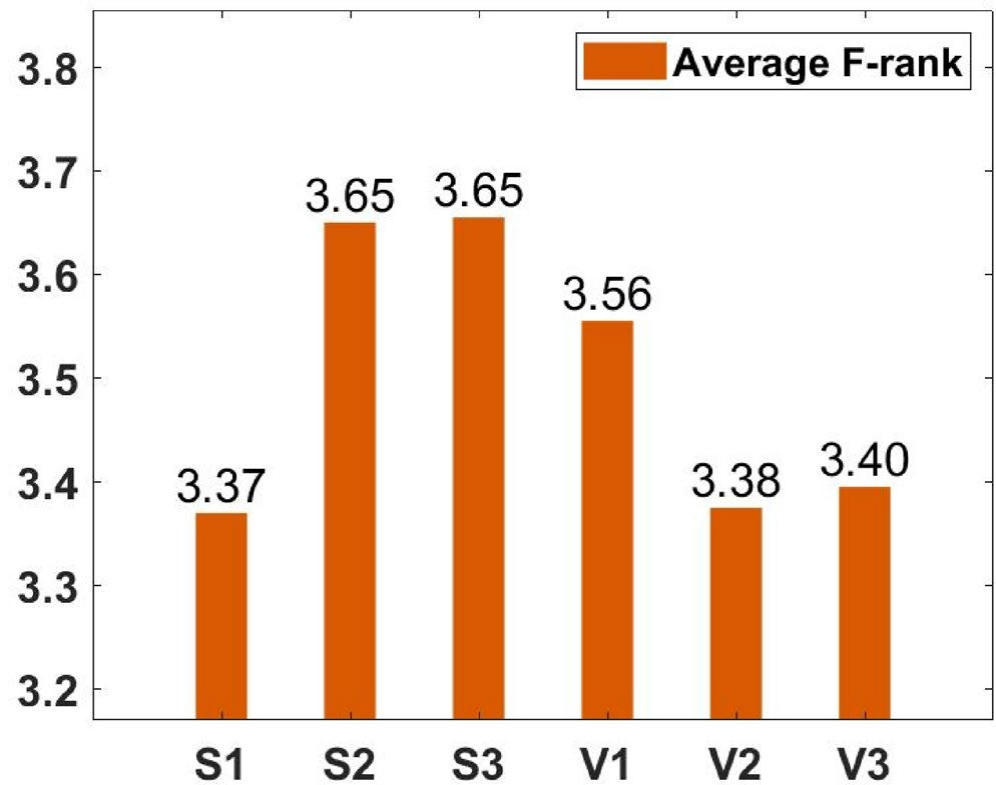
Average F-rank values for each TF.

The designed methodology introduces an encoding mechanism that integrates several TF to scale the encoded decision parameters so that their values fall between 0 and 1 bounds. Based on the normalized outcomes, a probabilistic process determines whether each UAV deployment point should be added, replaced, or retained in the population. In order to determine the optimal transfer function for the proposed FGODE algorithm, multiple functions belonging to both S-shaped and V-shaped families were examined through extensive experiments across 8 IoT devices instances. The results obtained, summarized in Table 8, and illustrated in Figs. 18, and 19, reveal that S1 offers more efficient accuracy compared with the other transfer functions according to AEC.

**Table 8:** Comparison of various transfer functions.

m		S1	S2	S4	V2	V3	V4
60	AEC	**1.077E + 06**	1.081E + 06	1.090E + 06	1.101E + 06	1.078E + 06	1.086E + 06
	F-rank	**3.16**	3.44	3.68	4.08	3.24	3.4
80	AEC	**1.566E + 06**	1.567E + 06	1.578E + 06	1.573E + 06	1.575E + 06	1.577E + 06
	F-rank	**3.24**	3.36	3.88	3.4	3.28	3.84
100	AEC	2.044E + 06	2.057E + 06	2.032E + 06	2.024E + 06	2.021E + 06	**2.021E + 06**
	F-rank	3.8	4.04	3.48	3.4	**3.24**	3.04
120	AEC	2.147E + 06	2.141E + 06	2.154E + 06	2.156E + 06	2.161E + 06	**2.135E + 06**
	F-rank	3.44	3.32	3.76	3.8	3.92	**2.76**
140	AEC	2.7794 + 06	2.791E + 06	2.767E + 06	2.779E + 06	**2.749E + 06**	2.767E + 06
	F-rank	3.56	4.12	3.48	3.64	**2.8**	3.4
160	AEC	**3.153E + 06**	3.158E + 06	3.193E + 06	3.159E + 06	3.165E + 06	3.167E + 06
	F-rank	**3.24**	3.32	4.04	3.6	3.48	3.32
180	AEC	3.572E + 06	3.587E + 06	3.577E + 06	3.561E + 06	3.578E + 06	3.589E + 06
	F-rank	3.28	3.8	3.4	**3.12**	3.6	3.8
200	AEC	**3.959E + 06**	3.975E + 06	3.970E + 06	3.962E + 06	3.962E + 06	3.976E + 06
	F-rank	**3.24**	3.8	3.52	3.4	3.44	3.6

**Values in bold** indicate the optimal results.

### Comparison between FGO and FGODE

This part focuses on analyzing the effectiveness of the enhanced mechanisms incorporated into the recently developed FGODE compared to the original FGO optimizer. The fitness outcomes of both algorithms are evaluated using the performance metrics considered after they are independently run 25 times. According to the reported results, FGODE consistently outperforms the standard FGO for all solved instances. This improvement is mainly attributed to the integration of DE, which strengthens exploitation and GM, which strengthens the exploration in FGODE and allows it to escape local optima more efficiently.

**Table 9:** Comparison between FGODE and FGO.

XXXX	FGODE			FGO			
	AEC	SD	FK	AEC	SD	FK	PV
60	**1.05E + 06**	**1.90E + 04**	**1.24**	1.08E + 06	2.77E + 04	1.76	**1.00E-03**
80	**1.56E + 06**	**3.40E + 04**	**1.24**	1.57E + 06	3.46E + 04	1.76	6.90E-02
100	**1.98E + 06**	**3.81E + 04**	**1.2**	2.04E + 06	6.21E + 04	1.8	**1.00E-03**
120	**2.11E + 06**	**4.23E + 04**	**1.4**	2.15E + 06	5.23E + 04	1.6	**2.10E-02**
140	**2.75E + 06**	**5.15E + 04**	**1.28**	2.7794 + 06	6.66E + 04	1.72	**3.70E-02**
160	**3.09E + 06**	**5.62E + 04**	**1.24**	3.15E + 06	6.42E + 04	1.76	**2.00E-03**
180	**3.55E + 06**	**5.78E + 04**	**1.28**	3.57E + 06	6.09E + 04	1.72	**4.50E-02**
200	**3.93E + 06**	6.95E + 04	**1.32**	3.96E + 06	**5.11E + 04**	1.68	**1.10E-02**

Values in **bold** indicate the optimal results.

**Fig. 20:**
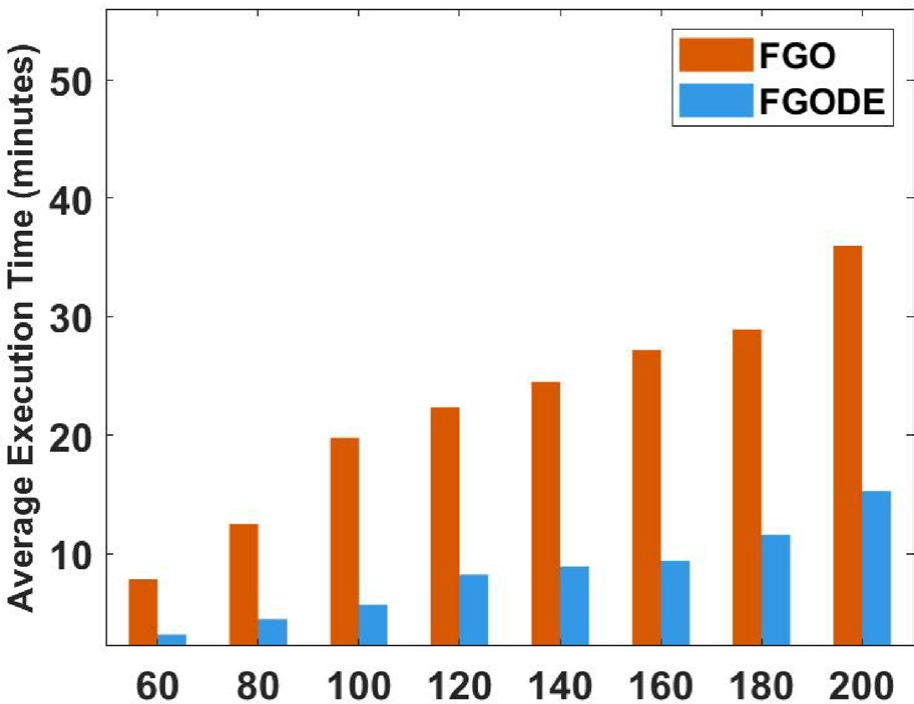
Average execution time of FGO and FGODE for each instance.

**Fig. 21:**
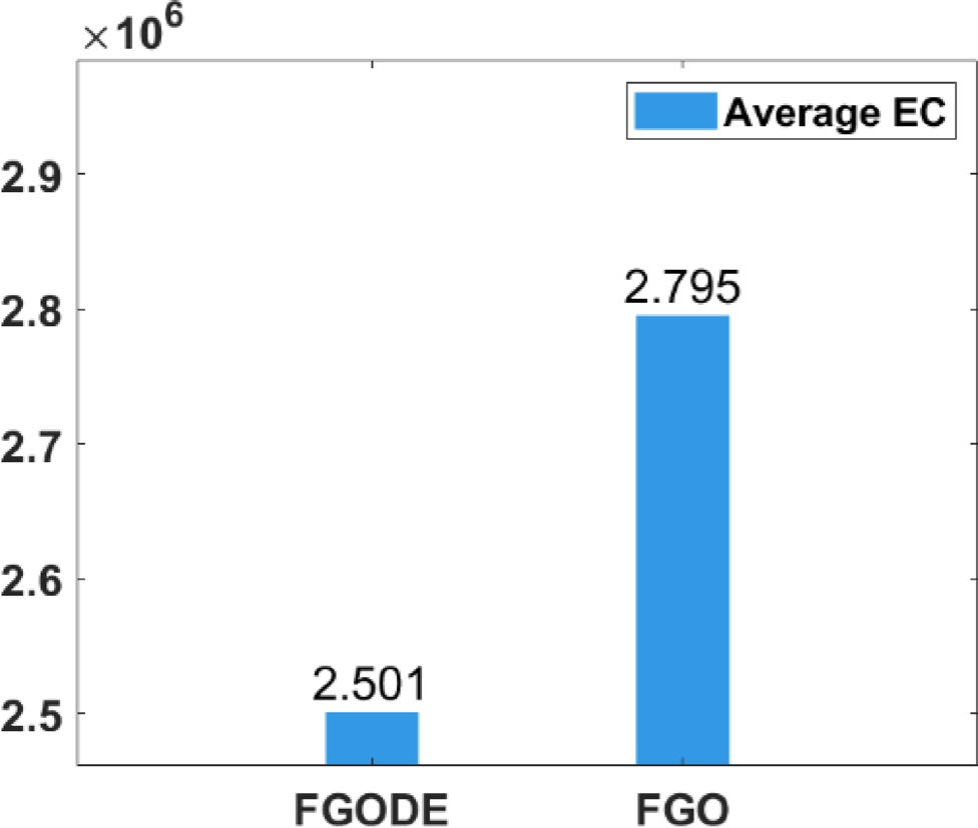
Average EC between FGODE and FGO.

**Fig. 22:**
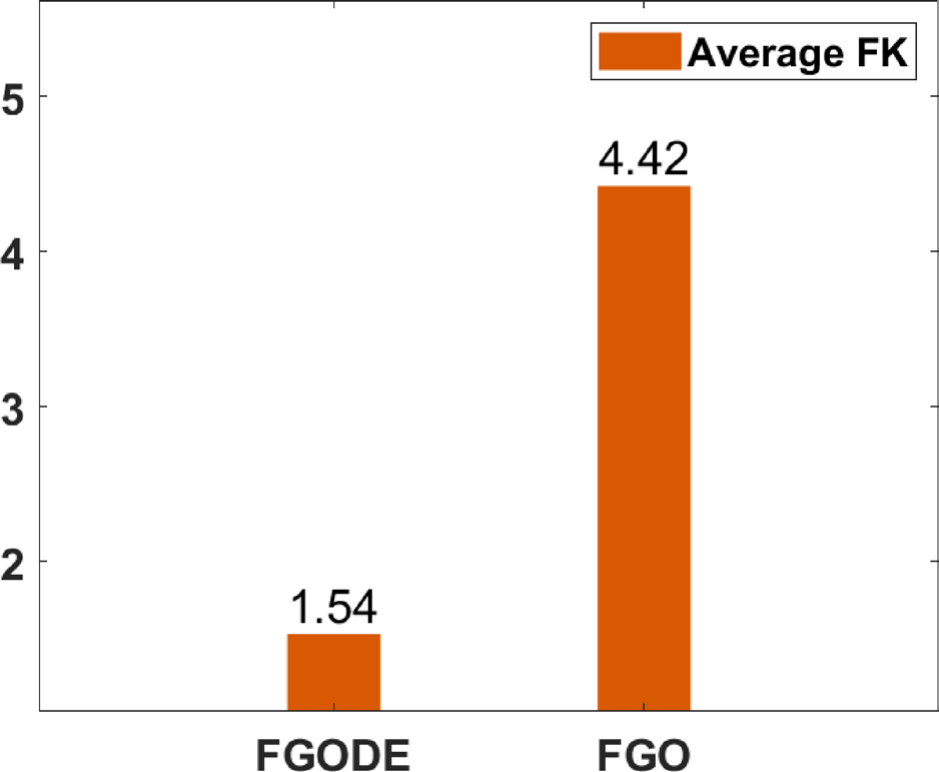
Average FK between FGODE and FGO.

**Fig. 23:**
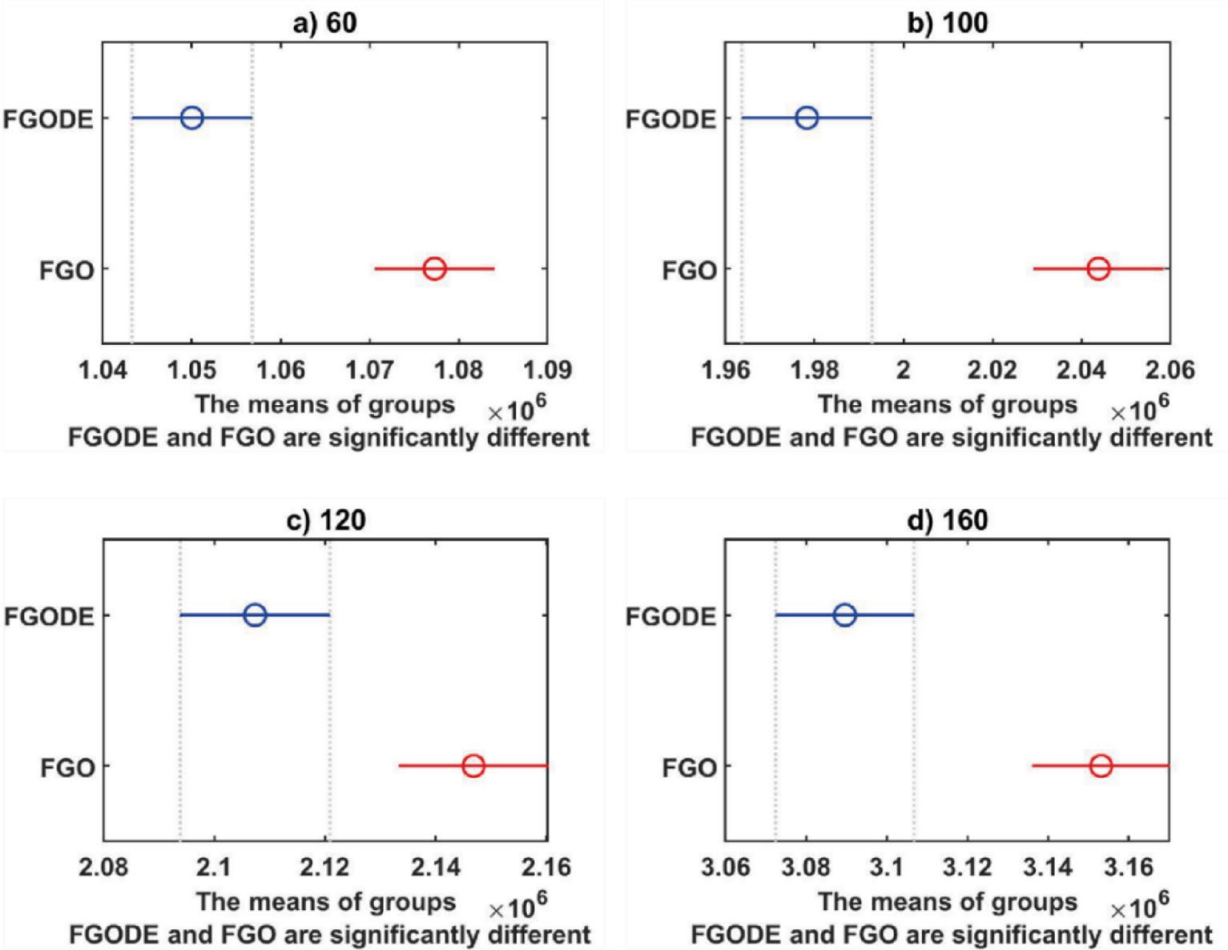
Performance comparison between FGODE and FGO using the FK-based multiple comparison test.

**Fig. 24:**
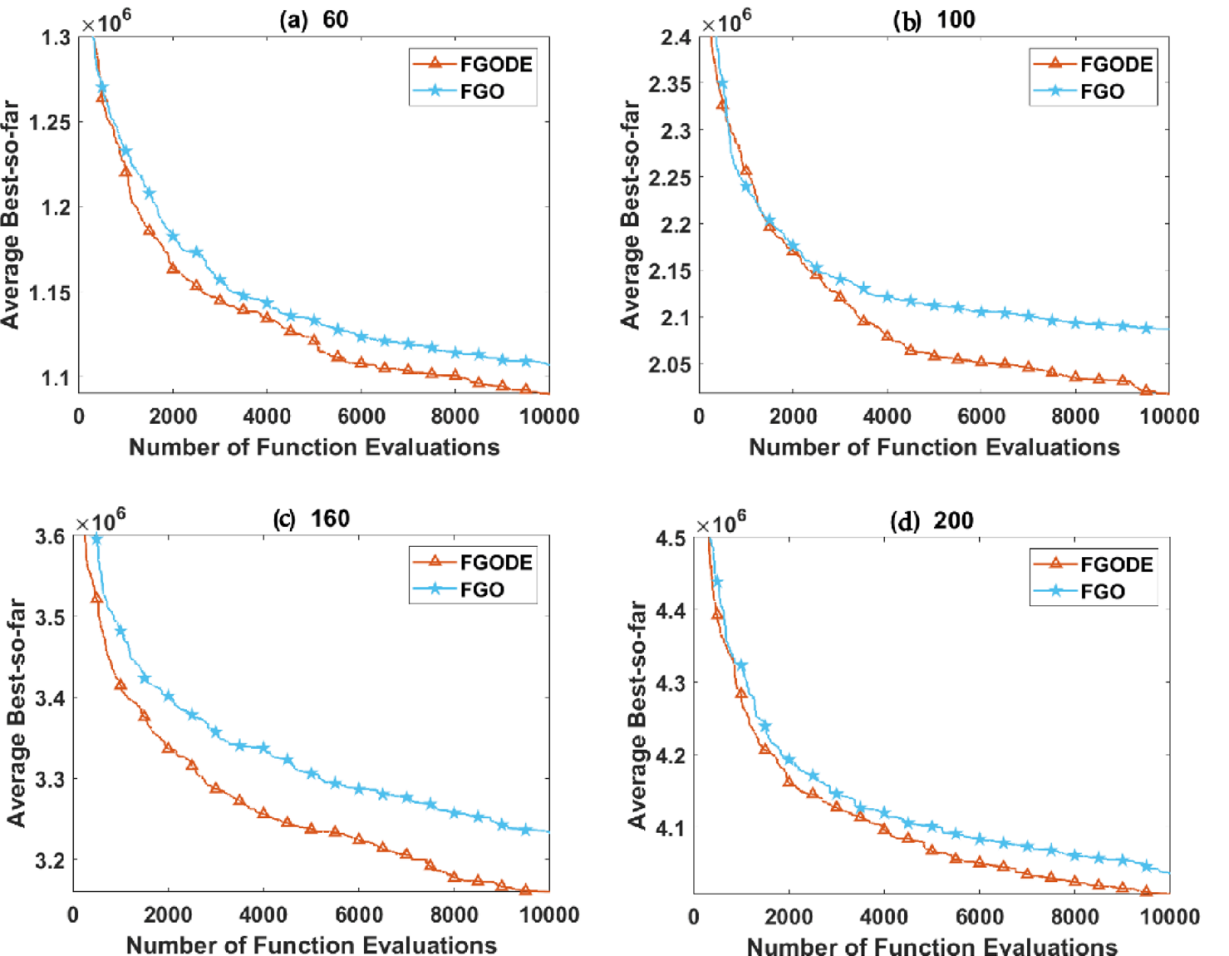
The convergence performance of the proposed FGODE and FGO in some instances.

In Table 9, the proposed FGODE attains better AEC and FK values than the FGO across all test cases and reaches outcomes that are significantly different, the WRS test is used, and its results PV values remain under 5% for all comparison instances except one instance, which shows a notable difference in outcomes produced by the two algorithms. Figure 20 shows the comparison of execution time between FGO and FGODE which reveals a noticeable difference in computational performance To simply illustrate the performance gap between FGO and FGODE, Fig. 21, shows the average AEC values, demonstrates that FGODE is the best-performing optimizer, achieving smaller energy consumption values. Figure 22, shows the difference between FGODE and FGO in average f-rank (FK), Fig. 23, shows that the means of the groups of the proposed FGODE and FGO are significantly different. Additionally, in Fig. 24, the convergence curves of both algorithms confirm that FGODE can reach the optimal region more rapidly, while FGO tends to converge slowly and sometimes get trapped in suboptimal regions. More generally, the statistical analysis reveals that FGODE produces quite different and better results compared to FGO for all solved instances.

In summary, this section’s experiments demonstrate that FGODE is a strong alternative to the original FGO, offering considerably improved solution quality, faster convergence behavior, and better consistency when tackling the UAV-assisted MEC optimization problem.

### Ablation study and component analysis

To further evaluate the contribution of each component in the proposed FGODE framework, an ablation study is conducted by developing several variants of the algorithm. The full model (FGODE), denoted as Version1 integrates all employed mechanisms, including DE, GM, LCG, and oPS. To analyze the impact of each component, three reduced variants are considered: FGODE without LCG (Version2), FGODE without GM (Version3), and FGODE without oPS (Version4), where each variant excludes one specific mechanism. In addition, the original Baseline FGO (Version5) is included for comparison without incorporating any enhancement mechanisms.

The performance of these variants is evaluated using AEC and F-rank. The obtained results, summarized in Table 10, clearly demonstrate that the full FGODE–Version1 consistently achieves the best performance among all variants. This confirms that each incorporated component contributes positively to the overall effectiveness of the proposed framework.

**Fig. 25:**
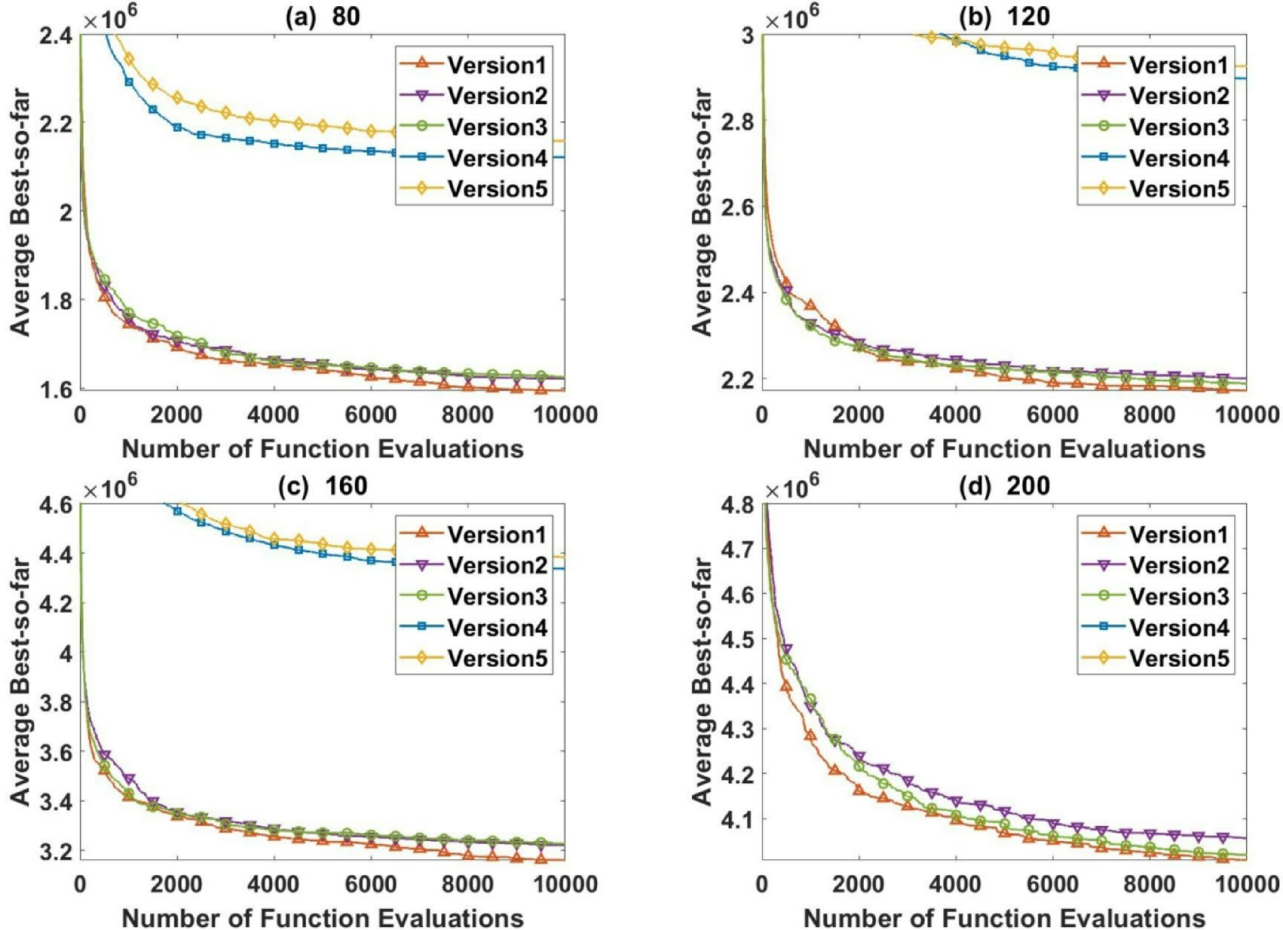
The convergence performance of several variants of the proposed FGODE in some instances.

Furthermore, convergence curves for selected problem instances are presented in Fig. 25, where FGODE–Version1 exhibits faster convergence and better stability compared to the other variants. These observations highlight the importance of combining the proposed mechanisms to achieve a balanced trade-off between exploration and exploitation, ultimately leading to superior optimization performance.

**Table 10:** Comparison of several variants of the proposed algorithm.

		Version1	Version2	Version3	Version4	Version5
60	**AEC**	**1.05E + 06**	1.08E + 06	1.07E + 06	1.40E + 06	1.46E + 06
	**F-rank**	**1.52**	2.4	2.08	4.36	4.64
80	**AEC**	**1.56E + 06**	1.59E + 06	1.59E + 06	2.06E + 06	2.14E + 06
	**F-rank**	**1.6**	2.2	2.2	4.16	4.84
100	**AEC**	**1.98E + 06**	2.04E + 06	2.04E + 06	2.68E + 06	2.78E + 06
	**F-rank**	**1.28**	2.32	2.4	4.36	4.64
120	**AEC**	**2.11E + 06**	2.16E + 06	2.15E + 06	2.83E + 06	2.91E + 06
	**F-rank**	**1.32**	2.4	2.28	4.32	4.68
140	**AEC**	**2.75E + 06**	2.77E + 06	2.77E + 06	3.71E + 06	3.80E + 06
	**F-rank**	**1.68**	2.16	2.16	4.28	4.72
160	**AEC**	**3.09E + 06**	3.15E + 06	3.17E + 06	4.26E + 06	4.36E + 06
	**F-rank**	**1.28**	2.16	2.56	4.36	4.64
180	**AEC**	**3.55E + 06**	3.56E + 06	3.56E + 06	4.85E + 06	4.97E + 06
	**F-rank**	**1.88**	2.12	2	4.4	4.6
200	**AEC**	**3.93E + 06**	3.98E + 06	3.95E + 06	5.37E + 06	5.54E + 06
	**F-rank**	**1.64**	2.2	2.16	4.16	4.84

Values in **bold** indicate the optimal results.

### Performance comparison of the proposed algorithms against recently developed algorithms

**Fig. 26:**
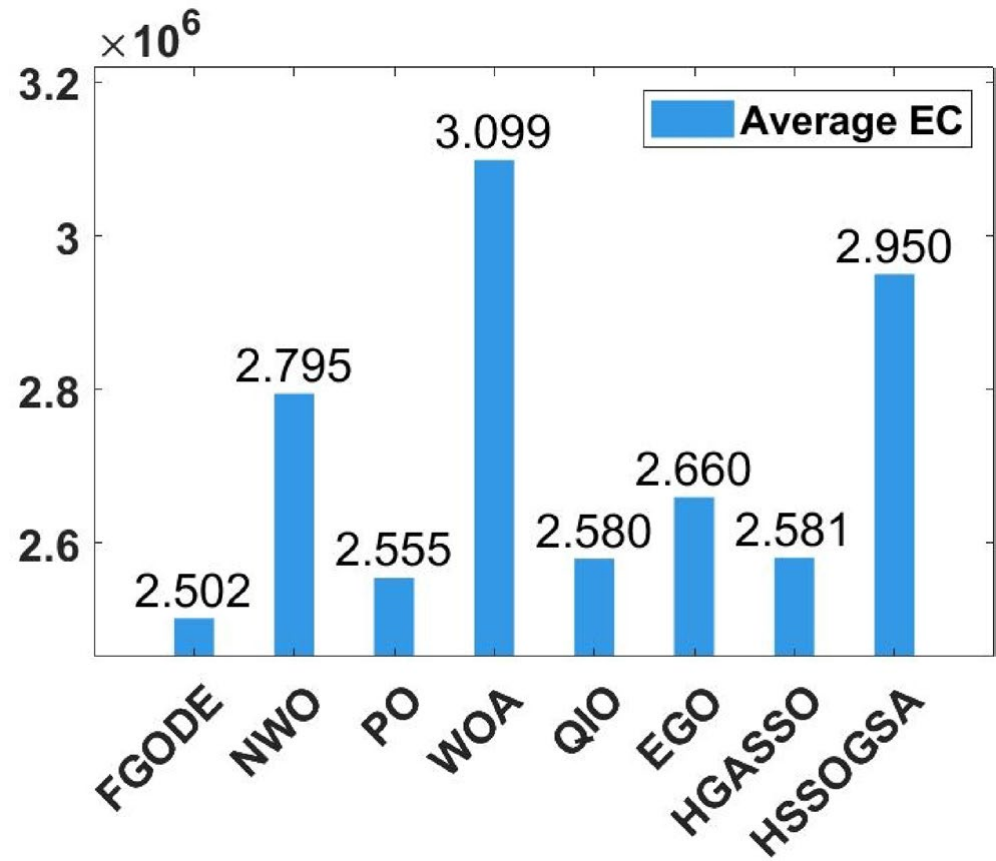
Comparison of average EC among several algorithmic models.

**Fig. 27:**
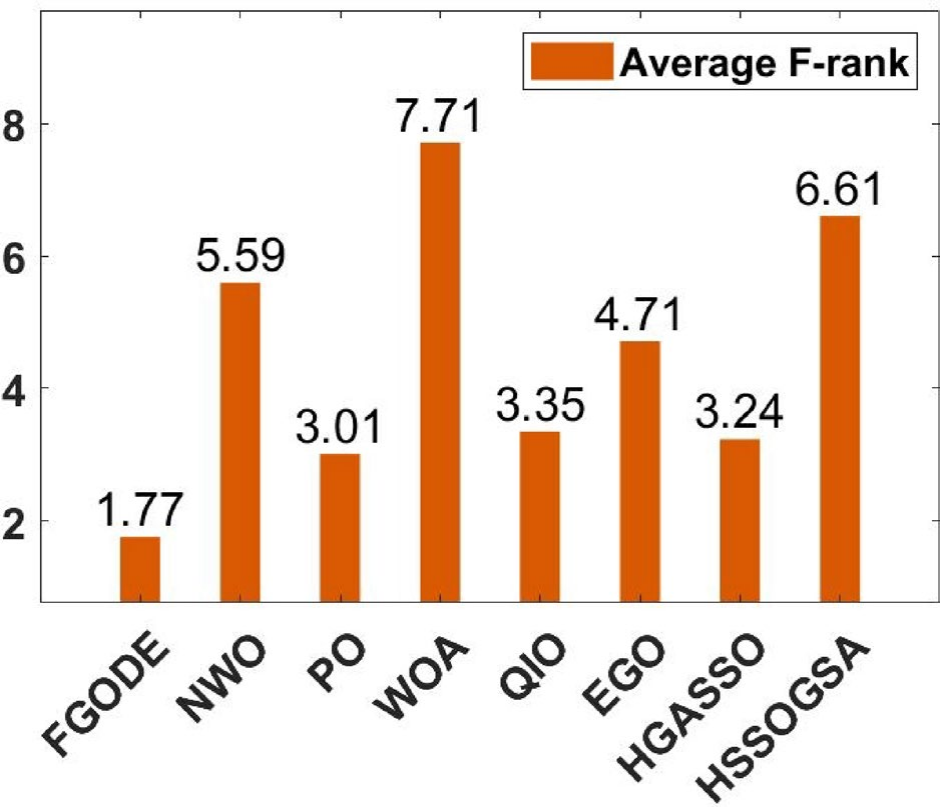
Comparison of average F-rank among several algorithmic models.

An evaluation of the proposed FGODE is conducted in comparison with seven recent trajectory planning algorithms. For every test instance, a total of 25 independent runs were conducted for each algorithm. FGODE ranks first across all evaluation indicators according to the data in Table 11. The statistical relevance of the observed differences is supported by the WRS test and its results PV, which are all below the 5% threshold. This validates the alternative hypothesis that the noted differences did not occur by random chance. To provide a clear comparative visualization, Fig. 26, illustrates AEC values attained by each algorithm across all evaluation scenarios. The figure confirms that FGODE obtains the lowest AEC (2.501E + 06), with PO ranking second (2.556E + 06). WOA again shows the highest (worst) average AEC, marking it as the least effective method. And Fig. 27, illustrates the average FK values and indicates that the proposed FGODE achieved the lowest FK rank among all compared algorithms.

**Fig. 28:**
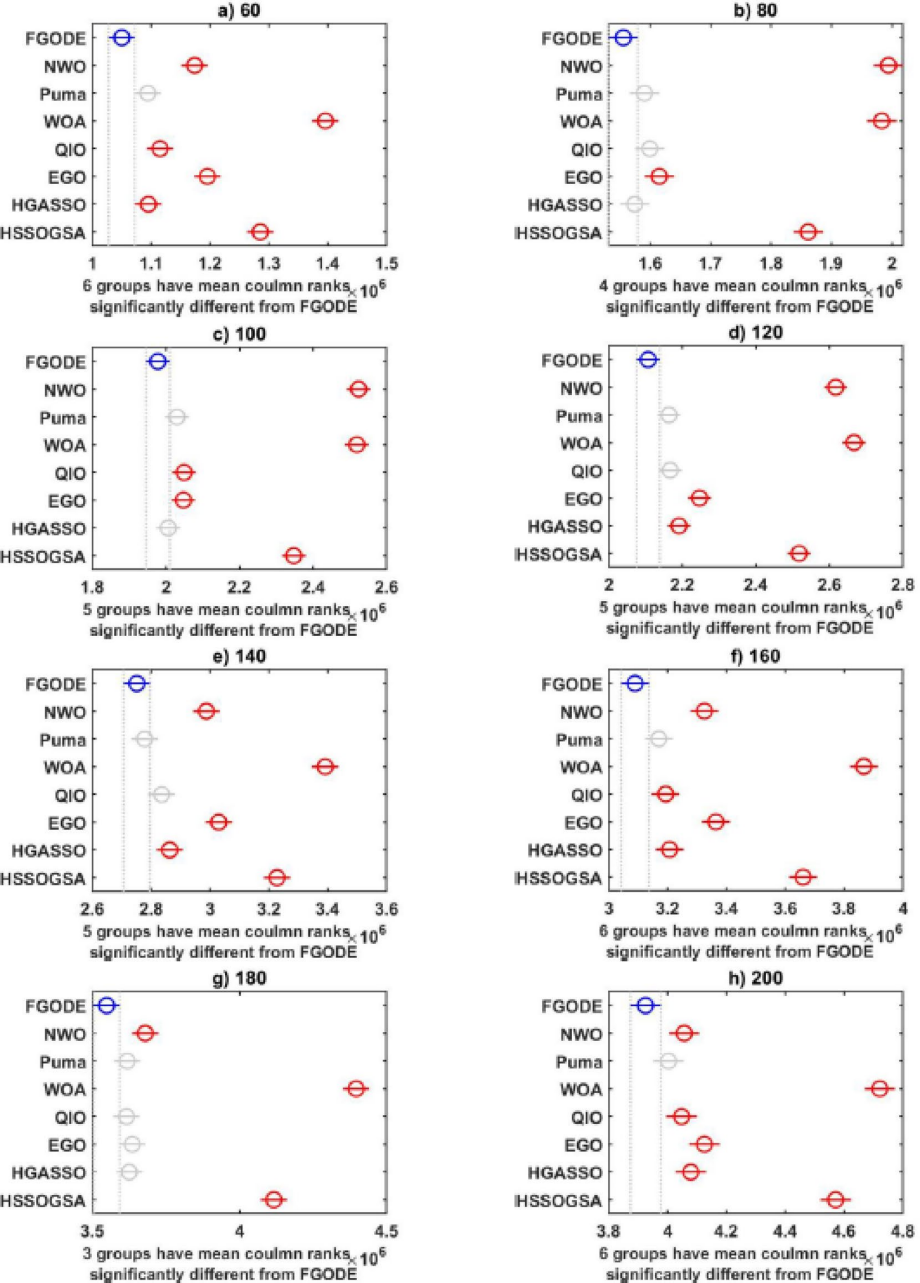
Performance comparison between FGODE and other metaheuristics using the FK-based multiple comparison test.

**Fig. 29:**
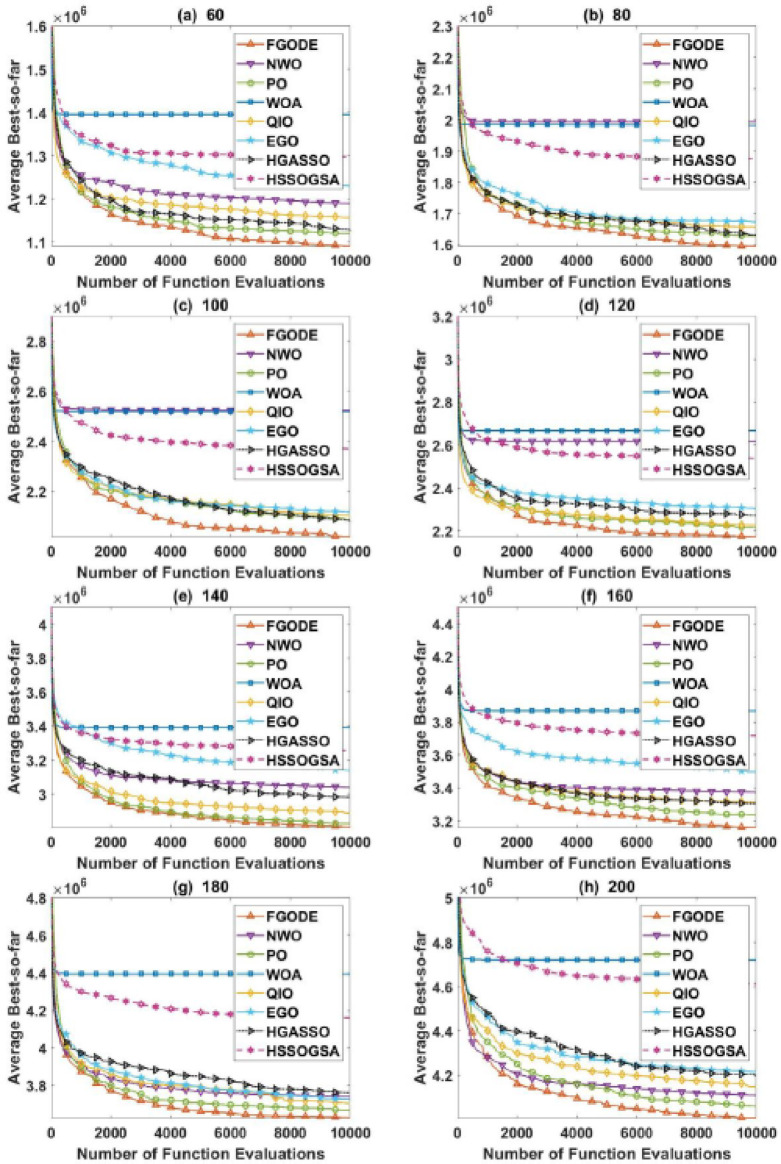
The convergence performance of the proposed FGODE alongside various metaheuristics.

To statistically validate the performance differences, an FK-based multiple comparison test was conducted, demonstrating that the mean performance of FGODE is significantly distinct from those of the competing algorithms. The experimental findings illustrated in Fig. 28. For the other scenarios, although a variation exists, its magnitude is relatively small. The convergence behavior of each algorithm is analyzed in Fig. 29, which plots the convergence curves for every test instance. These curves reveal which method reaches the minimum AEC value most rapidly. The analysis demonstrates that FGODE converges the fastest, PO is the second quickest, and WOA exhibits the slowest convergence speed. Collectively, the experimental findings in this section provide clear evidence that the proposed FGODE stands as a highly competitive alternative to recent trajectory-planning algorithms. These findings suggest that the mechanisms introduced demonstrate substantial improvement from the perspective of trajectory planning for multi-UAV-assisted MEC systems.

**Table 11:** Comparison between proposed algorithm FGODE and some recent algorithms.

	60				80			
	AEC	SD	FK	PV	AEC	SD	FK	PV
FGODE	**1.05E + 06**	**1.90E + 04**	**1.4**		**1.56E + 06**	**3.40E + 04**	**2.2**	
**NOW**	1.17E + 06	7.93E + 04	5	**1E-05**	1.99E + 06	7.22E + 04	7.36	**1E-05**
**PO**	1.09E + 06	3.40E + 04	2.92	**2E-05**	1.59E + 06	4.31E + 04	3.2	**2E-02**
**WOA**	1.40E + 06	5.21E + 04	7.96	**1E-05**	1.98E + 06	7.49E + 04	7.44	**1E-05**
**QIO**	1.12E + 06	5.10E + 04	3.44	**5E-05**	1.60E + 06	5.67E + 04	3.28	**2E-03**
**EGO**	1.20E + 06	4.69E + 04	5.48	**1E-05**	1.62E + 06	4.12E + 04	3.76	**1E-04**
**HGASSO**	1.10E + 06	3.57E + 04	3.2	**1E-05**	1.57E + 06	3.79E + 04	2.56	5E-02
**HSSOGSA**	1.29E + 06	7.02E + 04	6.6	**1E-05**	1.86E + 06	7.39E + 04	6.2	**1E-05**

	100				120			
	AEC	SD	FK	PV	AEC	SD	FK	PV
FGODE	**1.98E + 06**	3.81E + 04	**1.88**		**2.11E + 06**	**4.23E + 04**	**1.64**	
**NOW**	2.52E + 06	8.10E + 04	7.44	**1E-05**	2.62E + 06	1.04E + 05	7.04	**1E-05**
**PO**	2.03E + 06	3.63E + 04	3.36	**3E-04**	2.16E + 06	5.47E + 04	2.84	**1E-03**
**WOA**	2.52E + 06	9.31E + 04	7.32	**1E-05**	2.51E + 06	2.67E + 06	7.52	**1E-05**
**QIO**	2.05E + 06	7.59E + 04	3.4	**1E-03**	2.17E + 06	6.22E + 04	2.76	**3E-03**
**EGO**	2.05E + 06	**3.01E + 04**	3.96	**4E-05**	2.25E + 06	5.75E + 04	4.36	**1E-05**
**HGASSO**	2.01E + 06	6.64E + 04	2.56	**3E-02**	2.19E + 06	6.05E + 04	3.44	**1E-05**
**HSSOGSA**	2.35E + 06	1.27E + 05	6.08	**1E-05**	2.52E + 06	9.72E + 04	6.4	**1E-05**

	140				160			
	AEC	SD	FK	PV	AEC	SD	FK	PV
FGODE	**2.75E + 06**	**5.15E + 04**	**1.8**		**3.09E + 06**	**5.62E + 04**	**1.48**	
**NOW**	2.99E + 06	1.69E + 05	4.8	**3E-05**	3.33E + 06	1.43E + 05	4.8	**1E-05**
**PO**	2.78E + 06	6.33E + 04	2.28	2E-01	3.17E + 06	5.95E + 04	2.84	**2E-04**
**WOA**	3.39E + 06	1.37E + 05	7.88	**1E-05**	3.87E + 06	1.01E + 05	7.88	**1E-05**
**QIO**	2.84E + 06	7.39E + 04	3.24	**2E-04**	3.19E + 06	7.40E + 04	3.36	**1E-04**
**EGO**	3.03E + 06	6.91E + 04	5.76	**1E-05**	3.36E + 06	9.54E + 04	5.64	**1E-05**
**HGASSO**	2.86E + 06	9.10E + 04	3.36	**1E-05**	3.21E + 06	9.00E + 04	3.24	**1E-05**
**HSSOGSA**	3.23E + 06	1.24E + 05	6.88	**1E-05**	3.66E + 06	1.93E + 05	6.76	**1E-05**

	180				200			
	AEC	SD	FK	PV	AEC	SD	FK	PV
FGODE	**3.55E + 06**	**5.78E + 04**	**2.08**		**3.93E + 06**	6.95E + 04	**1.64**	
**NOW**	3.68E + 06	1.18E + 05	4.36	**1E-03**	4.06E + 06	1.08E + 05	3.96	**4E-04**
**PO**	3.62E + 06	5.90E + 04	3.64	**1E-03**	4.00E + 06	8.56E + 04	3.04	**2E-03**
**WOA**	4.40E + 06	1.39E + 05	7.92	**1E-05**	4.72E + 06	1.73E + 05	7.8	**1E-05**
**QIO**	3.62E + 06	8.39E + 04	3.52	**1E-02**	4.05E + 06	8.46E + 04	3.84	**2E-05**
**EGO**	3.64E + 06	6.37E + 04	3.8	**5E-05**	4.13E + 06	**6.21E + 04**	4.92	**1E-05**
**HGASSO**	3.63E + 06	7.97E + 04	3.72	**1E-05**	4.08E + 06	1.47E + 05	3.84	**1E-05**
**HSSOGSA**	4.12E + 06	1.69E + 05	6.96	**1E-05**	4.57E + 06	1.69E + 05	6.96	**1E-05**

Values in **bold** indicate the optimal results.

## Conclusion and future work

A new hybrid trajectory planning algorithm is introduced in this paper, namely FGODE, to effectively handle the UAV trajectory optimization task in UAV-assisted MEC systems. The developed algorithm combines the exploration quality of the Fungal Growth Optimizer (FGO) with the fast convergence ability of Differential Evolution (DE) to form a balanced hybrid search strategy. The first component utilizes FGO as the main optimization algorithm, mimicking the adaptive expansion behavior of fungal networks to explore diverse search regions. The second component integrates DE operators to enhance exploitation and accelerate convergence toward high-quality solutions.

Furthermore, an optimized Population Size-based encoding mechanism (oPS) is employed for an efficient representation of candidate solutions and improve diversity during the optimization process. To refine the UAV stop point trajectory, a low-complexity greedy (LCG) procedure is applied to minimize energy consumption while maintaining high coverage efficiency. Gaussian mutation (GM) is incorporated to enhance exploration by increasing variations in the population across the search environment. The population initialization stage employs a hybrid initialization strategy, which merges random generation, EOBL, and low-density region exploration (LDRE) to achieve a well-distributed and diverse starting population.

There are eight instances in which the proposed FGODE approach is tested to assess its performance metrics, and the number of IoT devices ranges from 60 to 200, representing small-to large-scale network environments. Multiple evaluation metrics such as average energy consumption, standard deviation, Friedman mean rank, Wilcoxon signed-rank test, and convergence speed, are used for the purpose of evaluating its efficiency. The findings from experiments verify that the developed hybrid FGODE algorithm exceeds performance of several cutting-edge competitors, achieving quicker attainment of optimality with improved solution precision and greater stability in determining optimal UAV trajectories for energy-efficient data collection in MEC-enabled IoT networks.

In future work, the proposed hybrid FGODE will be expanded to tackle joint task offloading and resource allocation challenges in extensive MEC systems. It will integrate multi-objective optimization and adaptive learning to improve scalability and **e**nable real-time decisions in dynamic IoT environments. Further theoretical analysis using complex network theory, sensitivity studies, and comparisons with new hybrid metaheuristics will be conducted to strengthen understanding and validation.

## Data Availability

All relevant data associated with this study may be requested directly from the corresponding author.
